# Structural and Biophysical Characterization of Purified Recombinant *Arabidopsis thaliana's* Alternative Oxidase 1A (rAtAOX1A): Interaction With Inhibitor(s) and Activator

**DOI:** 10.3389/fpls.2022.871208

**Published:** 2022-06-16

**Authors:** Tadiboina Veera Sankar, Moumita Saharay, Dharawath Santhosh, Abhaypratap Vishwakarma, Kollipara Padmasree

**Affiliations:** ^1^Department of Biotechnology and Bioinformatics, School of Life Sciences, University of Hyderabad, Hyderabad, India; ^2^Department of Systems and Computational Biology, School of Life Sciences, University of Hyderabad, Hyderabad, India; ^3^Department of Plant Sciences, School of Life Sciences, University of Hyderabad, Hyderabad, India; ^4^Department of Botany, Deshbandhu College, University of Delhi, New Delhi, India

**Keywords:** *Arabidopsis thaliana*, alternative oxidase, respiratory inhibitors, mutational docking, AOX structure, recombinant protein, circular dichroism, surface plasmon resonance

## Abstract

In higher plants, alternative oxidase (AOX) participates in a cyanide resistant and non-proton motive electron transport pathway of mitochondria, diverging from the ubiquinone pool. The physiological significance of AOX in biotic/abiotic stress tolerance is well-documented. However, its structural and biophysical properties are poorly understood as its crystal structure is not yet revealed in plants. Also, most of the AOX purification processes resulted in a low yield/inactive/unstable form of native AOX protein. The present study aims to characterize the purified rAtAOX1A protein and its interaction with inhibitors, such as salicylhydroxamic acid (SHAM) and n-propyl gallate (n-PG), as well as pyruvate (activator), using biophysical/*in silico* studies. The rAtAOX1A expressed in *E. coli* BL21(DE3) cells was functionally characterized by monitoring the respiratory and growth sensitivity of *E. coli*/pAtAOX1A and *E. coli*/pET28a to classical mitochondrial electron transport chain (mETC) inhibitors. The rAtAOX1A, which is purified through affinity chromatography and confirmed by western blotting and MALDI-TOF-TOF studies, showed an oxygen uptake activity of 3.86 μmol min^−1^ mg^−1^ protein, which is acceptable in non-thermogenic plants. Circular dichroism (CD) studies of purified rAtAOX1A revealed that >50% of the protein content was α-helical and retained its helical absorbance signal (ellipticity) at a wide range of temperature and pH conditions. Further, interaction with SHAM, n-PG, or pyruvate caused significant changes in its secondary structural elements while retaining its ellipticity. Surface plasmon resonance (SPR) studies revealed that both SHAM and n-PG bind reversibly to rAtAOX1A, while docking studies revealed that they bind to the same hydrophobic groove (Met191, Val192, Met195, Leu196, Phe251, and Phe255), to which Duroquinone (DQ) bind in the AtAOX1A. In contrast, pyruvate binds to a pocket consisting of Cys II (Arg174, Tyr175, Gly176, Cys177, Val232, Ala233, Asn294, and Leu313). Further, the mutational docking studies suggest that (i) the Met195 and Phe255 of AtAOX1A are the potential candidates to bind the inhibitor. Hence, this binding pocket could be a ‘potential gateway' for the oxidation-reduction process in AtAOX1A, and (ii) Arg174, Gly176, and Cys177 play an important role in binding to the organic acids like pyruvate.

## Introduction

The plant mitochondrial electron transport chain (mETC) possesses two terminal oxidases for the reduction of molecular oxygen into water (Rich and Moore, 1976). The cytochrome oxidase (COX) pathway is found in all eukaryotes. Electron transport through the COX pathway generates a proton gradient, which drives the ATP synthase for ATP production. The alternative oxidase (AOX) pathway is present in all plants, fungi, some protists, and a few animal species (Vanlerberghe and McIntosh, 1997; McDonald et al., 2009). AOX pathway, which branches at the ubiquinol pool, was identified during the thermogenic respiration studies on arum lilies (Meeuse, 1975; Rich and Moore, 1976). It is involved in a non-phosphorylating electron transport mechanism and dissipates excess energy as heat (Wagner and Moore, 1997; Siedow and Umbach, 2000; Millar et al., 2011; Moore et al., 2013). In higher plants, salicylhydroxamic acid (SHAM) and n-propyl gallate (n-PG) are frequently used to inhibit the activity of AOX, and, thereby, AOX pathway under both *in vitro* and *in vivo* conditions to reveal its physiological function(s) during normal growth, as well as biotic/abiotic stress conditions (Diethelm et al., 1990; Padmasree and Raghavendra, 1999a,b; Yoshida et al., 2006; Giraud et al., 2008; Dinakar et al., 2010; Florez-Sarasa et al., 2011; Zhang et al., 2011).

Mitochondrial AOX is encoded by a nuclear gene, and in many plants, it is known to exist as a multi-gene family and is regulated by retrograde signaling mechanisms (Whelan et al., 1996; Saisho et al., 1997; Liu and Butow, 2006; Rhoads and Subbaiah, 2007; Giraud et al., 2009; Ng et al., 2013). For instance, *Arabidopsis thaliana* has five AOX genes: *AOX1A-D* and *AOX2*. However, the isoforms corresponding to these genes cannot compensate for each other's function even under stress conditions (Considine et al., 2002; Clifton et al., 2006; Costa et al., 2017). A remarkable increase in the expression of AOX1A in *Arabidopsis thaliana* at a wide range of stress conditions and during the impairment of respiratory metabolism indicates its primary role in stress response as compared to other AOX genes (Clifton et al., 2005, 2006; Ho et al., 2008; Vishwakarma et al., 2015). In non-thermogenic plants, the AOX pathway is known to play a vital role in regulating the cellular redox balance when the cytochrome pathway is over-reduced or chemically inhibited and during abiotic stresses, such as high light, drought, temperature, UV-B stress, and high levels of greenhouse gasses (Berthold et al., 2000; Moore et al., 2002, 2013; Giraud et al., 2008; Vanlerberghe, 2013; Vishwakarma et al., 2014, 2015; Dahal and Vanlerberghe, 2017; Florez-Sarasa et al., 2020; Garmash et al., 2020). The AOX pathway is known to benefit plants by lowering ROS production, either by maintaining electron transport chain components upstream of the ubiquinone pool in a more oxidized state or by preventing the over-reduction of the COX pathway during stress conditions (Maxwell et al., 1999; Vishwakarma et al., 2015). The plants deficient in AOX1A showed acute sensitivity in response to light, drought, and antimycin A treatment than wild-type plants (Giraud et al., 2008). In contrast, overexpression of *AtAOX1A* induced tolerance to salt (Smith et al., 2009) and hypoxia (Vishwakarma et al., 2018) in *A. thaliana* plants. Similarly, plants lacking AOX were found to be more vulnerable to bacterial pathogens, sucking insects, and chewing herbivores as compared to wild-type plants (Zhang et al., 2012). Furthermore, infection with pathogens caused an increase in AOX transcript, as well as protein levels, thereby, AOX respiration in plants, substantiating the importance of AOX in biotic stress tolerance (Liao et al., 2012, 2021; Zhu et al., 2015).

The AOX is located on the inner membrane of mitochondria facing toward the mitochondrial matrix. Post-translational modifications in AOX, such as the disulfide bond formation, dimerization through non-covalent linkage, and its interaction with α-keto acids, such as pyruvate, are known to regulate its activity. The reduced form of AOX is found to be more active than the oxidized form as it interacts with α-keto acids through a thiohemiacetal linkage (Millar et al., 1993; Umbach and Siedow, 1993; Rhoads et al., 1998; Vanlerberghe et al., 1998; Umbach et al., 2002, 2006; Moore et al., 2013; Selinski et al., 2018; Xu et al., 2021). Besides, plant AOX isoforms expressed in *E. coli* membrane are known to be activated differently by 2-oxo acids/TCA cycle intermediates such as pyruvate, glyoxylate, citrate, oxaloacetate, malate, succinate, and α-ketoglutarate (Crichton et al., 2005, 2010; Carré et al., 2011; Ito et al., 2011; Moore et al., 2013; Selinski et al., 2016, 2017; Xu et al., 2021). Also, the addition of pyruvate is found to be essential to maintain the AOX activity during the harvesting of cells and purification of recombinant AOX from the *E. coli* membrane (Kido et al., 2010; Elliott et al., 2014).

Structural studies on the plant and trypanosomal AOX revealed that it possessed a non-heme diiron carboxylate active site (Berthold et al., 2000, 2002; Affourtit et al., 2002; Moore and Albury, 2008; Moore et al., 2008, 2013; Albury et al., 2009; Maréchal et al., 2009). The studies on the crystal structure of trypanosomal AOX (TAO) revealed that it exists as a homodimer and each monomer consists of a four-helix bundle (α2, α3, α5, and α6) ligated to a diiron core by highly conserved four glutamate and two histidine residues. Overall, there are six long and four short α-helices in each monomer, along with two hydrophobic cavities in TAO. The α1 and α4 helices of TAO were embedded in a large hydrophobic region that might be involved in membrane-binding. Furthermore, the binding of ubiquinol to Tyr220 of TAO leads to the catalysis of O_2_ reduction (Moore et al., 2013; Shiba et al., 2013; May et al., 2017). Besides, the studies of Elliott et al. (2014) revealed the secondary structure of a thermogenic rSgAOX, while such secondary structure is not yet unveiled for AOX from non-thermogenic plants. Also, despite the identification of several inhibitors and activators for AOX, the information on how these molecules interact with it is not yet known (Xu et al., 2021).

Several attempts have been made by various scientists to purify AOX from mitochondria of different plant sources (Huq and Palmer, 1978; Rich, 1978; Kay and Palmer, 1985; Bonner et al., 1986; Elthon and McIntosh, 1986, 1987; Berthold and Siedow, 1993; Zhang et al., 1996; Affourtit and Moore, 2004). However, the low yield and thermal instability of AOX impeded the studies related to its structural and biophysical properties. In this context, the rDNA technology allowed the expression of plant AOX in *E. coli*. The AOX from *A. thaliana* and Trypanosoma were expressed in a ΔhemA *E. coli* strain, which is found to be deficient in the cytochrome pathway (Kumar and Söll, 1992; Nihei et al., 2003). Besides, the supplementation of iron in the form of Fe^2+^/Fe^3+^ was in ambiguity during heterologous expression of AOX to acquire it in pure and active form (Minagawa et al., 1990; Ajayi et al., 2002; Affourtit and Moore, 2004).

Thus, this study aims to acquire an adequate amount of AtAOX1A pure protein by expressing it in a routinely used laboratory strain [BL21(DE3)] of *E. coli* and understand its secondary structure and stability toward temperature and pH. Also, the present study intends to examine the interaction of AtAOX1A with well-known inhibitors (SHAM and n-PG) and activators (pyruvate) under *in vitro* conditions by employing CD spectroscopy, SPR, and molecular docking studies. Besides, respiratory and growth inhibitory studies were performed in *E. coli* transformed with AtAOX1A to ensure its functional expression.

## Materials and Methods

### Reagents and Chemicals

Sodium pyruvate, tris base, imidazole, duroquinone, diethyl ether, sodium dithionite, n-dodecyl β-D maltoside (DDM), octyl-β-D glucopyranoside (OG), isopropyl β-D-1-thiogalactopyranoside (IPTG), phenylmethylsulfonyl fluoride (PMSF), n-PG, SHAM, coomassie brilliant blue R-250, and TRIzol reagent were purchased from Sigma Aldrich, St Louis, Mo, USA. The protease inhibitor ‘cocktail' was purchased from Roche. CM5 sensor chips, PBS, surfactant P20, glycine-HCl pH 2.5, sodium acetate pH 5, and amine coupling kit were purchased from GE Healthcare Bio-Sciences Corp., USA. Verso cDNA synthesis kit, 50 bp DNA ladder, bicinchoninic acid (BCA) protein estimation kit, protein molecular mass standard, and 3 kDa cut-off snakeskin dialysis membrane were purchased from Thermo Fisher Scientific, USA. Talon cobalt metal affinity resin was purchased from Clontech, Takara Bio, Japan. His-Tag mouse monoclonal antibody (HRP conjugated) was purchased from Cell Signaling Technologies. ECL Western blotting reagents were purchased from GE Healthcare Bio-Sciences Corp., USA. Polymerase chain reaction (PCR) components and restriction endonucleases were purchased from New England Biolabs. Luria-Bertani medium, agar-agar, and kanamycin were purchased from Hi-Media, Mumbai, India. Ferrous sulfate and glycerol were purchased from SRL. All the compounds used were of the biochemical grade.

### Strains and Plasmid Construction

The *Escherichia coli* (*E. coli*) strain DH5α was used to maintain the clone, while the strain BL21(DE3) (InvitrogenTM, Waltham, MA, USA) was used for the expression of recombinant protein AOX, which has a His_6_-tag on it. Further details on cloning of AtAOX1A and plasmid construction are described in Vishwakarma et al. (2016).

### Method of Measuring Cell Growth

The BL21(DE3) cells transformed with pET28a (*E. coli*/pET28a) and recombinant pET28a with AtAOX1A (*E. coli*/pAtAOX1A) were inoculated into 3 ml of LB medium containing 50 μg/ml of kanamycin and incubated overnight at 37°C (pre-culture). The pre-culture was inoculated into 20 ml of LB medium with 50 μg/ml of kanamycin and allowed to grow. As the OD_600_ of the culture reached 0.15, IPTG (0.1 mM) was added into the culture medium to induce rAtAOX1A. To ascertain the functions of rAtAOX1A in *E. coli*, the cultures were treated with each of the following mitochondrial inhibitors at a wider range of concentrations: 0.05–1 mM KCN (inhibitor of cytochrome oxidase pathway), 0.025–0.5 mM n-PG and 0.5–2 mM SHAM (inhibitors of alternative oxidase pathway). Subsequently, both control (*E. coli*/pET28a) and transformed (*E. coli*/pAtAOX1A) cells were incubated for 5 h at 37°C, and OD_600_ was monitored at regular intervals of 30 min (Kumar and Söll, 1992; Berthold, 1998; Fukai et al., 1999).

### Assay of Cell Respiration

After induction of rAtAOX1A with IPTG as described above, *E. coli* were extracted and suspended in a suitable volume of LB medium to achieve a cell density of 20 at OD_600_. The cellular respiration of both *E. coli*/pET28a and *E. coli*/pAtAOX1A were measured in terms of rates of oxygen consumption with or without the addition of KCN, n-PG, and SHAM. Respiration rates were measured with a total of 4.8 × 10^8^ cells/ml for each reaction using the Clark-type oxygen electrode (Kirimura et al., 1999; Ajayi et al., 2002; Oxygraph plus, Hansatech instruments, UK).

### Preparation of *E. coli*/pAtAOX1A Membrane Sample

The *E. coli* carrying recombinant AtAOX1A was pre-cultured overnight at 37°C in 10 ml of LB medium containing 50 μg/ml of kanamycin. About 5 ml of pre-culture was inoculated into 500 ml of LB medium containing 50 μg/ml kanamycin and 0.1 mM ferrous sulfate and grown at 37°C with 200 rpm. The culture was allowed to grow until OD_600_ reached 0.4 and IPTG (0.1 mM) was added to induce the expression of rAtAOX1A. Subsequently, the culture was allowed to grow at 28°C for 4 h, and cells were harvested by centrifugation at 5,000 rpm for 7 min at 4°C. The pellet obtained was resuspended in 50 mM Tris-HCl containing 10 mM pyruvate at pH 7.5.

*E. coli* cells were lysed by sonication with an amplitude of 35 for 20 min by switching on/off of the probe at regular intervals of 30 s each, in the presence of a protease inhibitor cocktail and PMSF (1 mM). After lysis, cell debris was removed in a single step by centrifugation at 12,000 g. The supernatant was centrifuged at 200,000 g for 1 h at 4°C to separate cytoplasm from membrane fraction. The membrane pellet was resuspended in a minimal volume of 50 mM Tris-HCl (pH 7.5) containing 10 mM of pyruvate.

### Solubilization of rAtAOX1A From *E. coli* Membranes

The solubilization (10 ml) buffer, which contains 1% (w/v) DDM in 50 mM of Tris-HCl, along with 10 mM of pyruvate and 20% (v/v) glycerol at pH 7.5, was used to solubilize the membrane fraction obtained from *E. coli*/pAtAOX1A as described above. The buffer was added dropwise with gentle mixing and immediately centrifuged for 1 h at 200,000 g. All the steps were performed at 4°C. The supernatant enriched in rAtAOX1A is labeled as DDM extract.

### Purification of rAtAOX1A

Purification of rAtAOX1A was done according to Kido et al. (2010) and Elliott et al. (2014), with minor modifications. The cobalt resin (selective for His-tag) was equilibrated with an equilibration buffer at a 1:3 ratio for 1 h. The equilibration buffer contained 0.5% (w/v) DDM and 0.5% (w/v) OG in 50 mM of Tris-HCl (pH 7.5), along with 10 mM of pyruvate, 20% (v/v) glycerol, and 100 mM of MgSO_4_. After equilibration, 10 ml of DDM extract was added to the resin and allowed to mix gently overnight at 4°C. Further, the resin which is bound with rAtAOX1A was washed with 10 ml of wash buffer, which contained 0.5% (w/v) DDM and 0.5% (w/v) OG in 50 mM Tris-HCl (pH 7.5), along with 10 mM of pyruvate, 20% (v/v) glycerol, 100 mM MgSO_4_, and 50 mM of imidazole. Subsequently, the resin was transferred onto a column and rAtAOX1A was eluted as 1-ml fractions using elution buffer, which contained 0.5% (w/v) DDM and 0.5% (w/v) OG, in 50 mM of Tris-HCl along with 10 mM of pyruvate, 250 mM of imidazole, 20% (v/v) glycerol, and 100 mM of MgSO_4_ at pH 7.5. Protein purification was visualized on 12.5% SDS-PAGE under reducing conditions, as described in Laemmli (1970). The gel was stained using Coomassie Brilliant Blue and the protein was estimated using the BCA kit.

### Western Blot Analysis

The proteins from sodium dodecyl sulfate-polyacrylamide gel electrophoresis (SDS-PAGE) were electrophoretically transferred onto the polyvinylidene difluoride (PVDF) membrane using a Western blotting unit (Smart Scientific Instruments, Chennai), as described by Towbin et al. (1979). After transfer, the PVDF membrane was incubated at 4°C with a mouse monoclonal anti-His antibody (1:5,000 dilution) for 5–6 h, which is conjugated with horseradish peroxidase (Cell signaling technology). The blot was washed with tris buffered saline containing 0.2% of tween 20 (TBST) for 30–40 min. The blot was developed using Enhanced chemiluminescence (ECL) western blotting reagents and rAtAOX1A protein was detected with the help of the ChemiDoc imaging system (BioRad).

### Oxygen Uptake Activity

The activity of rAtAOX1A was measured polarographically by monitoring the oxygen uptake rates using an S1 Clark-type oxygen electrode (Oxygraph plus, Hansatech Instruments, UK). The activity assay was performed by adding 0.7 μg of purified protein in 400 μl of air saturated reaction medium (50 mM of Tris-HCl, pH 7.5), and the reaction was started by the addition of 600 μM DQH_2_ as a substrate. The stocks of DQH_2_ were prepared on the day of use by suspending the solid DQH_2_ in acidified ethanol (Rich, 1978). The stock concentration of DQH_2_ was determined based on its molar extinction coefficient (2.15 mM^−1^ cm^−1^ at 283 nm in water) using a UV-Visible spectrophotometer (Shimadzu UV-1700). The acidified ethanol blank was subtracted from the assay while measuring the activity.

### MALDI TOF/TOF Analysis

The purified recombinant protein band was excised with a sharp and sterile scalpel from the gel after performing SDS-PAGE and kept in a 1.5-ml tube. Later, the gel plug was reduced with DTT (10 mM) and alkylated with iodoacetamide (55 mM) before subjecting to digestion with trypsin (12.5 μg/μl). The tryptic digested protein sample was analyzed by using matrix-assisted laser desorption ionization time-of-flight mass spectrometry (MALDI-TOF MS). The method uses Autoflex III smart beam instrument (Bruker Daltonics, Bremen, Germany), equipped with a nitrogen laser (355 nm), and operated in a reflection mode for peptide mass and sequencing in the presence of α-cyano-4-hydroxycinnamic acid as a matrix, as described in Swathi et al. (2014). The spectra from MALDI-MS and MALDI-MS-MS ionization were searched with the MASCOT search engine. The biotools software (Bruker Daltonics, version 3.1) was used to analyze the lift spectra, while the Sequence similarity analysis was performed by the National Center for Biotechnology Information (NCBI) blast tool.

### Circular Dichroism (CD) and Thermal Stability Studies

The purified rAtAOX1A present in the elution buffer described above was transferred into a 10-mM phosphate buffer (pH 7.5) through the process of step-down dialysis for performing CD. Three different dialysis buffers [**Buffer 1**: 20 mM Tris-HCl (pH 7.5) containing 10 mM of pyruvate, 0.03% of DDM, and 10% of Glycerol; **Buffer 2**: 10 mM of phosphate buffer (pH 7.5) containing 0.03% of DDM and 1% of glycerol and **Buffer 3**: 10 mM of phosphate buffer (pH 7.5)] were used at a ratio of 10:500 (sample volume to buffer volume). The sample was dialyzed in each buffer for a period of 4 h at 4°C under continuous stirring. Finally, the rAtAOX1A present in 10 mM of phosphate buffer (pH 7.5) was concentrated by lyophilization. The CD spectrophotometer (Jasco J-1500, Japan) coupled with a thermostat was used to analyze rAtAOX1A (0.8 mg/ml). The parameters used for the CD scan were as follows: wavelength, 190 to 260 nm; speed, 50 nm/min; step resolution, 1 nm; bandwidth, 1 nm; response, 3 s; temperature, 25°C, and sensitivity, 50 mdegrees. Similarly, the secondary structural stability of rAtAOX1A at different pH, from 2 to 12, was determined after incubating rAtAOX1A in different buffers [5 mM of Glycine-HCl (pH 2), 10 mM of sodium phosphate (pH 7.5)—control sample and 5 mM Glycine-NaOH (pH 10-12)] at 37°C for 1 h. Conversely, for the temperature melting studies, the parameters used were as follows: wavelength, 190 to 260 nm; response, 1 s; temperature, 4 to 90°C; speed, 50 nm/min; and sensitivity, 50 mdegrees (Kelly et al., 2005; Elliott et al., 2014). Furthermore, different concentrations (0.05, 0.1, and 0.5 mM) of SHAM and n-PG were added separately to 0.4 mg/ml of purified rAtAOX1A to analyze the secondary structure conformational changes induced in rAtAOX1A during its interaction with the inhibitors. Similarly, different concentrations (0.05, 0.1, 0.5, and 1 mM) of pyruvate was added to 0.4 mg/ml of purified rAtAOX1A to study the secondary structure conformational changes induced in rAtAOX1A during its interaction with the activator. The changes in secondary structural elements of rAtAOX1A were analyzed by submitting the raw CD data to the online server named Dichroweb (Whitmore and Wallace, 2008) using the CDSSTR algorithm with a reference set 4 (Sreerama and Woody, 2000).

### Surface Plasmon Resonance (SPR) Studies

The purified rAtAOX1A protein sample was dialyzed into a 10-mM phosphate buffer (pH 7.5) by using above-mentioned step-down dialysis procedure and concentrated by lyophilization.

#### Immobilization of the rAtAOX1A on CM5 Sensor Chip

The following steps are performed to immobilize the rAtAOX1A on the CM5 sensor chip: (i) a mixture of 40 mM of 1-ethyl-3-(3-dimethylaminopropyl) carbodiimide (EDC) and 10 mM of N-hydroxysuccinimide (NHS) are passed through the reference (blank) flow cell (Fc-3), as well as the sample flow cell (Fc-4) of CM5 sensor chip at a flow rate of 30 μl/min for 300 s, to generate reactive succinimide esters; (ii) the rAtAOX1A (100 μg/ml) prepared in sodium acetate buffer (pH 5) was injected into the activated flow cell (Fc-4), where the NHS esters spontaneously react with uncharged amino groups of rAtAOX1A (ligand) and covalently link it to the dextran matrix; (iii) the residual active NHS esters in Fc-4 are blocked by passing 1 M of ethanolamine-HCl (pH 8.5) for 300 s, at a flow rate of 30 μl/min; (iv) all the active NHS esters of the reference (blank) flow cell (Fc-3), which is devoid of rAtAOX1A are blocked with ethanolamine-HCl (pH 8.5).

#### Sample Injection

The analytes SHAM and n-PG (1 to 5 mM) dissolved in double distilled water are injected independently into the flow cells (Fc-3 & Fc-4) in a running buffer composed of PBS (2 mM of KH_2_PO_4_, 10 mM of Na_2_HPO_4_, 2.7 mM of KCl, and 137 mM of NaCl at pH 7.4) containing 0.005% P20 surfactant, at a flow rate of 30 μl/min at 25°C. In the association phase (120 s), the analytes were allowed to bind to the rAtAOX1A, while in the dissociation phase (120 s), they are separated from each other. In the regeneration phase (30 s), the analytes are disposed of from the flow cells using 10 mM of Glycine-HCl (pH 2.5) and 0.5 M of NaCl, as described in the Biacore T-200 manual (GE Healthcare Life Sciences). The experiment was performed twice. In each experiment, three cycles of association, dissociation, and regeneration were carried out against each concentration of inhibitors (SHAM and n-PG) tested in the present study.

#### Kinetic Analysis

The acquired data were analyzed using BIA evaluation software (version 2.0, GE Healthcare Life Sciences) with the Langmuir fit model of 1:1 binding. To determine the kinetic constants (*k*_a_, *k*_d_, and *K*_D_), the sensorgrams were fitted to a 1:1 kinetic model using the pooled data analysis option. The *k*_a_ represents the association constant, *k*_d_ represents the dissociation constant, and *K*_D_ represents the equilibrium dissociation constant. The affinity of inhibitor(s) with rAtAOX1A was derived by measuring the kinetic parameters at five different concentrations (1, 2, 3, 4, and 5 mM) of n-PG and SHAM.

### Molecular Docking

The homology model of the AtAOX1A monomer (PMDB Accession number: PM0080189) was chosen as the target protein for the docking study (Pennisi et al., 2016). The 3-dimensional models of the ligands ubiquinol-1 (Q_1_H_2_), ubiquinone-1 (UQ_1_), duroquinol (DQH_2_), duroquinone (DQ), SHAM, n-PG, and pyruvate were generated using PubChem (Kim et al., 2021) and CHARMM-GUI (Jo et al., 2008) programs. The binding affinities of different ligands, such as Q_1_H_2_, UQ_1_, DQH_2_, DQ, SHAM, n-PG, and pyruvate with AtAOX1A are studied using the SwissDock program (Grosdidier et al., 2011a,b). To identify the potent residues that may have a direct impact on the binding of the ligands (DQ, SHAM, n-PG, and pyruvate), point mutations with alanine were performed.

### Statistical Analysis

The data shown are the Mean ± SE of three replicates. The statistical differences were evaluated by one-way ANOVA with the Tukey test integrated into Sigma plot, version 12.0, Systat Software Inc., San Jose, CA, USA, at a significance level of *P* ≤ 0.05.

## Results

### Cloning and Expression of *Arabidopsis thaliana AOX1A* (*AtAOX1A*)

**Figure 1:**
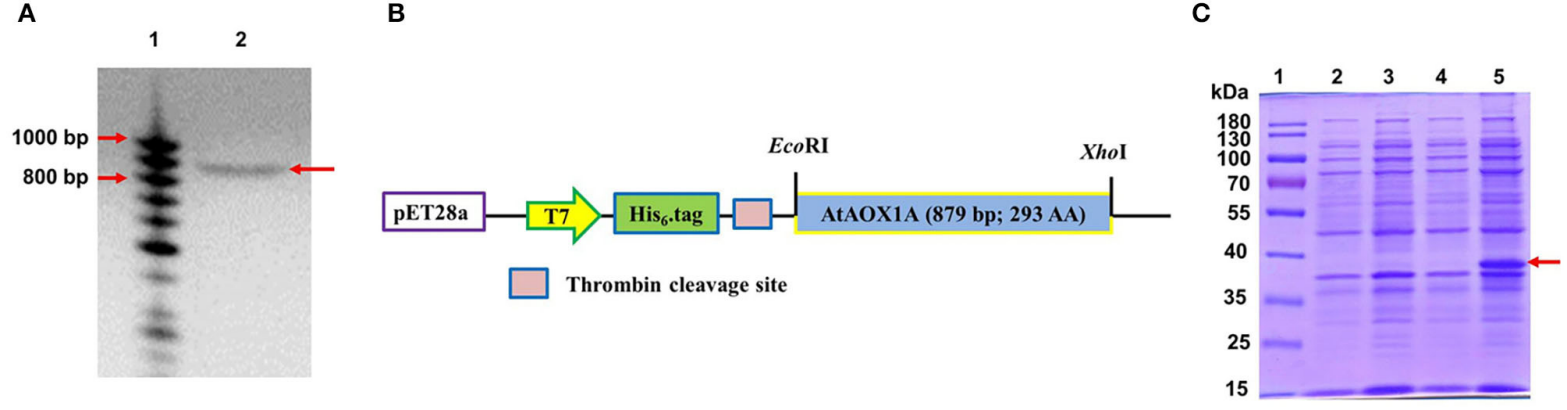
Cloning and expression of *AtAOX1A* in *E. coli***. (A)** Polymerase chain reaction (PCR) amplification of 879 bp *AtAOX1A* using gene-specific primers. Lane 1, 50–1000 bp DNA ladder; lane 2, PCR amplified *AtAOX1A* (indicated with arrow). **(B)** Schematic diagram of the expression vector construction. The gene sequences were cloned into restriction endonuclease *Eco*RI and *Xho*I recognition sites of the pET28a vector to produce N-terminal His_6_-tag recombinant AtAOX1A protein. **(C)** Total protein of both *E. coli*/pET28a and *E. coli*/pAtAOX1A cells were separated on 12.5% (w/v) sodium dodecyl sulfate-polyacrylamide gel electrophoresis (SDS-PAGE) for recombinant protein expression analysis. Each well was loaded with 40 μg of protein. Lane 1, 10–180 kDa protein marker; lane 2, *E. coli*/pET28a; lane 3, *E. coli*/pET28a induced with 0.1 mM IPTG; lane 4, *E. coli*/pAtAOX1A; lane 5, *E. coli*/pAtAOX1A induced with 0.1 mM IPTG. The induced rAtAOX1A is indicated with an arrow.

The complementary DNA (cDNA) of *AtAOX1A* coding for mature protein was amplified (Figure 1A) using *AOX1A*- specific primers (Vishwakarma et al., 2016) and cloned into a pET28a vector (Figure 1B). The pAtAOX1A construct was transformed into BL21(DE3) cells. Transformed positive colonies (*E. coli*/pAtAOX1A) were selected by colony PCR (Supplementary Figure 1A). Recombinant plasmids from these colonies were isolated and confirmed by restriction digestion with *Eco*RI and *Xho*I (Supplementary Figure 1B) and DNA sequencing using T7 primers (Supplementary Data S1). After confirming the *AtAOX1A* sequence, these colonies were used for recombinant protein synthesis. The expression of rAtAOX1A protein (~37 kDa) in *E. coli*/pAtAOX1A after induction with IPTG was visualized on 12.5% (w/v) SDS-PAGE under reducing conditions (Figure 1C).

### Functional Characterization of rAtAOX1A in *E. coli*

**Figure 2:**
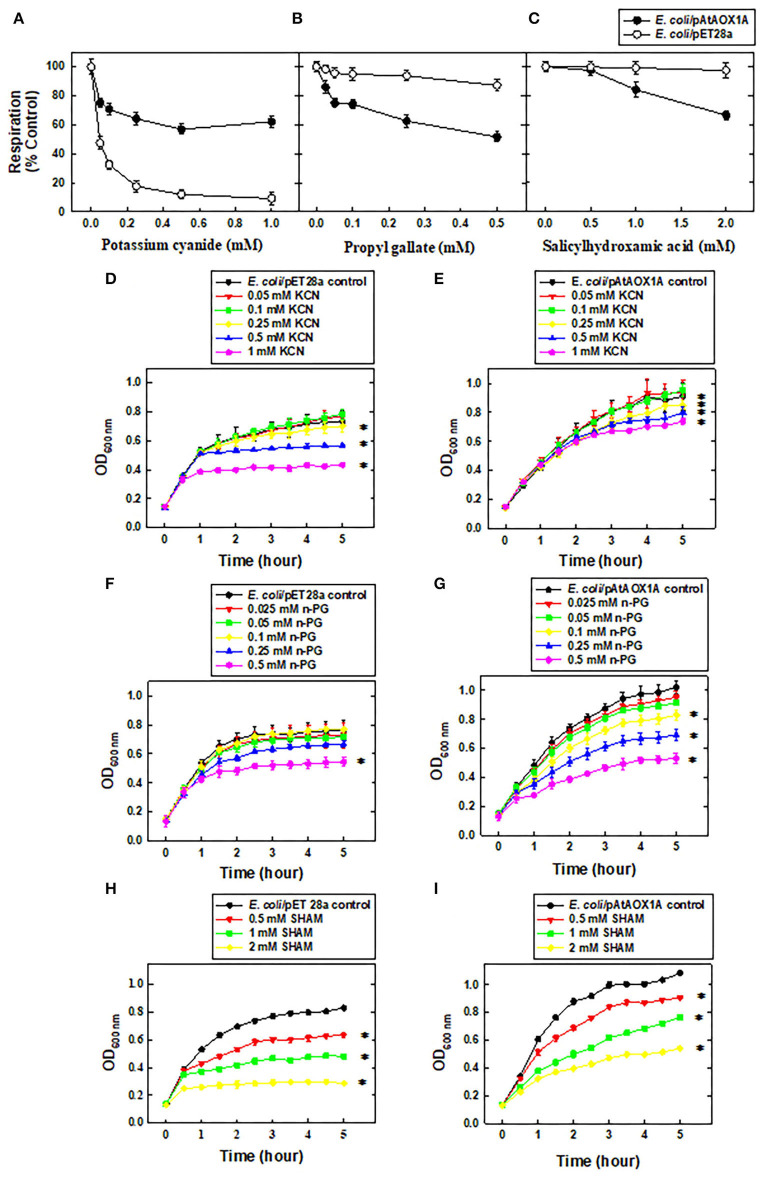
Functional characterization of rAtAOX1A in *E. coli*. Oxygen uptake rates (%) of *E. coli*/pET28a and *E. coli*/pAtAOX1A in the absence or presence of respiratory inhibitors: **(A)** KCN (0.05, 0.1, 0.25, 0.5, and 1 mM), **(B)** n-PG (0.025, 0.05, 0.1, 0.25, and 0.5 mM), and **(C)** SHAM (0.5, 1, and 2 mM). The growth pattern of *E. coli* cells was monitored for 5 h in the absence or presence of respiratory inhibitors: **(D)** Growth pattern of *E. coli*/pET28a and **(E)** *E. coli*/pAtAOX1A in the absence or presence of KCN; **(F)** Growth pattern of *E. coli*/pET28a and **(G)** *E. coli*/pAtAOX1A in the absence or presence of n-PG; **(H)** Growth pattern of *E. coli*/pET28a and **(I)** *E. coli*/pAtAOX1A in the absence or presence of SHAM. Each value represents the mean ± SD of three experiments. The statistical significance difference (*P* <0.05) was calculated for the endpoint of the growth curve and indicated with asterisks.

To identify that expressed rAtAOX1A is functionally active, both *E. coli*/pET28a and *E. coli*/pAtAOX1A were evaluated for their respiratory and growth rates in the presence of KCN, n-PG, and SHAM at a wide range of concentrations (Figure 2). In the absence of any inhibitor, both *E. coli*/pET28a (34.52 ± 0.5 μmoles O_2_ min^−1^) and *E. coli*/pAtAOX1A (32 ± 0.74 μmoles O_2_ min^−1^) showed comparable respiratory O_2_ uptake rates per 4.8 x 10^8^ cells ml^−1^. However, supplementation of KCN at an increasing concentration from 0.05 to 1 mM caused a significant reduction in the O_2_ uptake (up to 90%) of *E. coli*/pET28a due to inhibition of COX-catalyzed respiration, whereas it only caused 37% reduction in respiratory rate of *E. coli*/pAtAOX1A, suggesting that remaining O_2_ uptake in transformed cells is contributed by AOX-catalyzed respiration (Figure 2A).

In contrast to KCN, supplementation of n-PG, a commonly used inhibitor of AOX, has shown only a marginal (<13%) effect on respiratory O_2_ uptake rates of *E. coli*/pET28a even at a concentration as high as 0.5 mM due to the lack of AOX-catalyzed respiration. Conversely, at this concentration, n-PG caused a 50% reduction in respiratory rates of *E. coli*/pAtAOX1A (Figure 2B). In the case of SHAM supplementation, another inhibitor of the AOX respiratory pathway decreased the respiratory rates of *E. coli*/pAtAOX1A remarkably up to 33%, as its concentration is increased from 0.5 to 2 mm. However, treatment of *E. coli*/pET28a with 2 mM SHAM caused only a 2.5% reduction in its respiratory rate (Figure 2C).

To examine the effect of respiratory inhibitors on the growth of the *E. coli*/pET28a and *E. coli*/pAtAOX1A, the bacterial growth was compared at the end of 5 h. Interestingly, *E. coli*/pET28a achieved a stationary phase after 4 h (Figures 2D,F,H), while *E. coli*/pAtAOX1A cells remained in a log phase even after 5 h of growth (Figures 2E,G,I), suggesting the role of AOX-catalyzed respiration in enhancing the metabolic activities of *E. coli*. The supplementation of KCN at an increasing concentration (0.05 to 1 mM) has suppressed the growth of *E. coli*/pET28a significantly by 51%. However, KCN has suppressed only 23% of growth in *E. coli*/pAtAOX1A (Figures 2D,E). When *E. coli*/pET28a was supplemented with n-PG, it caused only a trivial (14%) effect on its growth even at 0.25-mM concentration, whereas it suppressed the growth of *E. coli*/pAtAOX1A up to 38% at the same concentration. However, n-PG at higher concentration (0.5 mM) showed remarkable growth inhibitory effect on both *E. coli*/pET28a (34%) and *E. coli*/pAtAOX1A (54%), respectively (Figures 2F,G). On contrary, SHAM has suppressed the growth of *E. coli*/pET28a and *E. coli*/pAtAOX1A by 88 and 57%, respectively, as its concentration was raised from 0.5 to 2 mM (Figures 2H,I). However, the greater effect of SHAM on the growth of *E. coli*/pET28a, as compared to *E. coli*/pAtAOX1A, might be due to the non-specific inhibitory effect on other oxidases of *E. coli*, besides its specific effect on AOX. These results, taken together, suggest that rAtAOX1A expressed in *E. coli*/pAtAOX1A is functionally active.

### Purification of rAtAOX1A and Its Analysis by MALDI TOF/TOF

**Figure 3:**
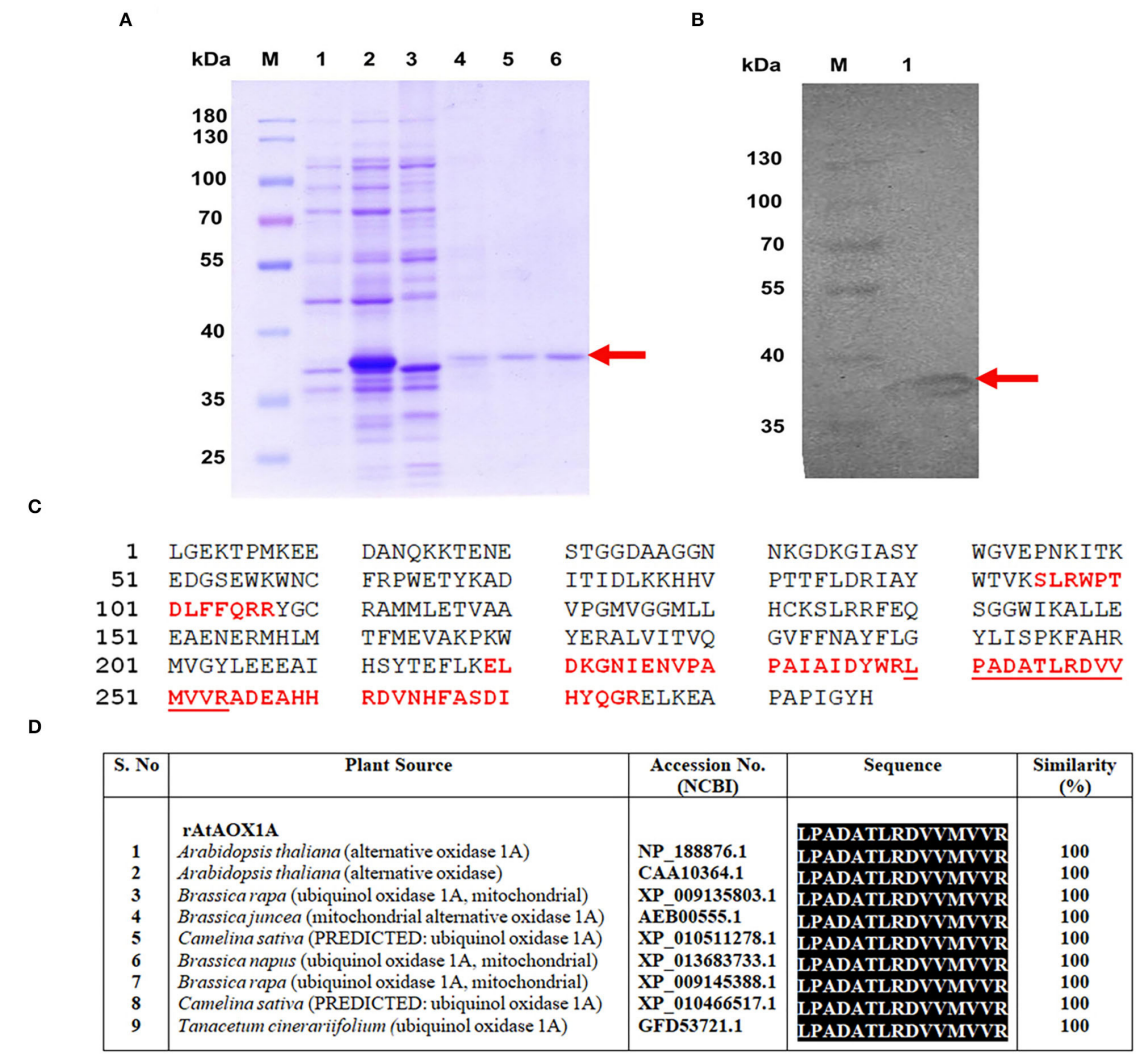
Purification and validation of rAtAOX1A protein using MALDI-MS-MS. **(A)** SDS-PAGE visualizing purification profile of rAtAOX1A from *E. coli*. Lane M, 10-180 kDa protein marker; lane 1, protein from *E. coli*/pAtAOX1A; lane 2, protein from *E. coli*/pAtAOX1A induced with IPTG; lane 3, flow-through; lane 4, washing fraction; lanes 5 and 6, elution fractions, arrow indicates purified rAtAOX1A with ~37 kDa molecular mass. **(B)** Western blot showing purified rAtAOX1A: lane M, 10-180 kDa protein marker; lane 1, 40 μg of purified rAtAOX1A. **(C)** *A. thaliana* partial AOX (gi/1872517) sequence from NCBI database. The sequence of MS peaks (1209.854, 1365.972, 1566.099, 1656.258, 2385.628, and 2476.697 Da) of rAtAOX1A are indicated in bold red font, and sequence coverage from these six major peaks is about 24%. The obtained sequence from peptide peak m/z 1656.258 (‘LPADATLRDVVMVVR') has shown 100% identity with AtAOX1A in BLAST search **(D)**. The gel picture shown is the selective representation of three biological replicates.

As the expression cassette contains an N-terminal His_6_-tag, cobalt column affinity chromatography was used to purify rAtAOX1A from the soluble fraction of *E. coli*/pAtAOX1A membrane. To get active protein: (1) rAtAOX1A was induced by IPTG in the presence of FeSO_4_ (0.1 mM) and (2) after induction, the harvested cells were suspended in 50 mM Tris-HCl containing 10 mM of pyruvate at pH 7.5. The purified rAtAOX1A with a molecular mass of ~37 kDa was observed in reducing SDS-PAGE, which includes AtAOX1A mature protein (33.44 kDa) and pET28a (+) vector sequence (3.83 kDa) (Figure 3A). Furthermore, western blot analysis with anti-His antibody showed a single band correlating with the molecular mass observed in SDS-PAGE (Figure 3B). The rAtAOX1A has shown a specific activity of 3.86 μmol O_2_ min^−1^ mg^−1^ protein and a 15% recovery rate, where 600 μM of duroquinol was used as a substrate (Table 1).

**Table 1:** The membrane fractions of *E. coli* were prepared from 500 ml of *E. coli*/pAtAOX1A culture and used for rAtAOX1A purification.

**Fraction**	**Total activity (μmol O_**2**_ min^**−1**^)**	**Protein yield (mg/500 ml culture)**	**Specific activity (μmol O_**2**_ min^**−1**^ mg^**−1**^ protein)**	**Recovery (%)**
*E. coli*/pAtAOX1A lysate	14.57	44.17	0.33	100
Inner membrane	29.95	19.2	1.56	43.4
DDM extract	20.76	17.16	1.21	39.8
Purified rAtAOX1A	25.47	6.6	3.86	14.94

*Oxygen uptake activities were measured using an oxygen electrode in the presence of duroquinol as a substrate*.

*The specific activity shown here is a representative of three independent experiments*.

Moreover, the purified recombinant protein band (~37 kDa) from SDS-PAGE (Figure 3A) was digested with trypsin and subjected to MALDI-TOF/TOF analysis. The MS-MS ion search in the Mascot search database resulted in matching with partial *A. thaliana* AOX (ID: gi/1872517) with a significant score of 308 (Supplementary Data S2). The six major peptide peaks (m/z 1209.854, m/z 1365.972, m/z 1566.099, m/z 1656.258, m/z 2385.628, and m/z 2476.697) in peptide mass fingerprint (PMF) spectrum covered 24% of partial *A. thaliana* AOX (ID: gi/1872517) protein sequence from NCBI database (Figure 3C). The Biotools software revealed the following sequence ‘LPADATLRDVVMVVR' for lift spectra corresponding to the peak 1656.258 Da (Supplementary Figure 2). Blast search of the sequence (LPADATLRDVVMVVR) showed 100% identity with the *A. thaliana* AOX1A in the NCBI database (Figure 3D). These results confirm that the purified protein is rAtAOX1A.

### CD Spectroscopic Analysis of rAtAOX1A and Its Interaction With SHAM, n-PG, and Pyruvate

The CD spectroscopy is frequently used to study the secondary structure and its conformational state in proteins. It detects the difference in the absorption of left and right-handed circularly polarized light in optically active molecules such as proteins, and differences in the absorbance are monitored in terms of ellipticity. In the CD spectroscopy graph, a combination of a particular wavelength of positive and negative peaks defines the secondary structure, such as: (1) A positive peak at 192 nm and negative peaks at 208 and 222 nm represent an α-helical structure; (2) A positive peak at 195 nm, and a negative peak at 216 nm indicates a β-sheet structure; (3) A positive peak at 205 nm represents a β-turn, and (4) A negative peak at 200 nm and a positive peak at 212 nm represent a random coil (Kelly and Price, 2000; Kelly et al., 2005).

**Figure 4:**
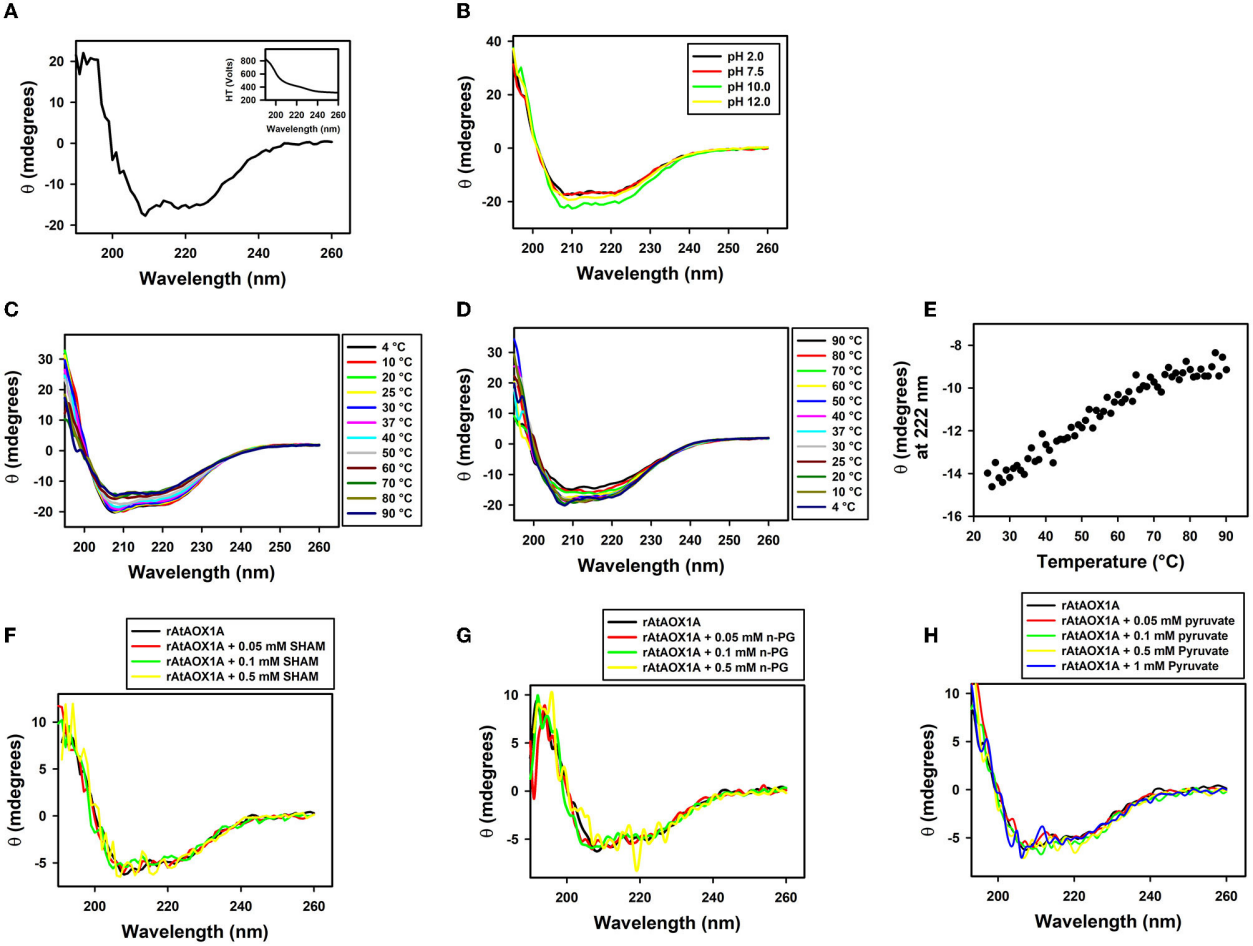
Secondary structural stability analysis of rAtAOX1A. **(A)** CD spectra of purified rAtAOX1A at far UV (190-260 nm) in 10-mM phosphate buffer. Ellipticity in θ (mdeg) of rAtAOX1A (0.8 mg/ml) was plotted against the wavelength. Inset: High Tension profile of voltage trace. The secondary structural stability was determined at different **(B)** pH from 2 to 12; **(C)** Temperature from 4 to 90°C; **(D)** Temperature from 90 to 4°C. **(E)** Ellipticity of rAtAOX1A at 222 nm between 25°C and 90°C. **(F)** CD spectra of rAtAOX1A at far UV (190–260 nm), with 0.05, 0.1, and 0.5 mM SHAM and, without inhibitor; **(G)** CD spectra of rAtAOX1A at far UV (190-260 nm), with 0.05, 0.1, and 0.5 mM n-PG, and without inhibitor; **(H)** CD spectra of rAtAOX1A at far UV (190-260 nm), with 0.05, 0.1, 0.5, and 1 mM of pyruvate, and without activator. Concentration of purified rAtAOX1A used to obtain the CD spectrum (SHAM, n-PG, and pyruvate) was 0.4 mg/ml. The final spectrum is an average of three scans as described in the Materials and Methods section.

The secondary structural analysis of rAtAOX1A in Far-UV (190–260 nm) CD spectra indicated that it possesses an α-helical structure (Figure 4A). The presence of two negative peaks at 208 nm and 222 nm is a characteristic feature of the typical α-helical structure. The high tension (HT) voltage trace was shown as an inset in Figure 4A. Further, any change in the pH (2–12) caused only a marginal variation in the ellipticity of rAtAOX1A, as compared to the ellipticity observed at optimal pH 7.5 (Figure 4B). Similarly, the thermal treatment of rAtAOX1A showed only a marginal decrease in its ellipticity with a gradual increase in the temperature from 4° to 90°C (Figure 4C). However, this effect was reversed by gradually cooling down the temperature from 90° to 4°C (Figure 4D), suggesting that rAtAOX1A is structurally stable to heat treatment. The axes for spectra in Figures 4B–D are enlarged between 205 to 230 nm to bring more clarity between different treatments (Supplementary Figures 3A–C). Also, the protein has retained its helical absorbance signal (222 nm) up to ~67% despite an increase in temperature from 25° to 90°C (Figure 4E). These results suggest that rAtAOX1A is highly stable at a wide range of temperature and pH conditions.

Furthermore, the alterations in secondary structural elements of purified rAtAOX1A upon addition of SHAM, n-PG, and pyruvate were determined separately by CD spectroscopy. The addition of different concentrations of SHAM, n-PG, or pyruvate to rAtAOX1A has not shown any deviation in the helical absorbance signal in the CD spectrum observed between 190–260 nm. However, a marginal deviation in the negative peaks was observed (Figures 4F–H). The axes for spectra in Figures 4F–H are enlarged between 205 and 230 nm to bring more clarity between different treatments (Supplementary Figures 3D–F). Further, the changes in the secondary structure composition of the rAtAOX1A in the presence of SHAM, n-PG, and pyruvate were summarized in Table 2. The addition of both inhibitors (SHAM and n-PG) and activator (pyruvate) to the rAtAOX1A caused an increase in β-sheets (~7%) with a concomitant decrease in α-helical (~10%) content, while the changes in β-turns and random coils (together) are negligible (~3%).

**Table 2:** Modulation in rAtAOX1A secondary structural elements during interaction with SHAM, n-PG, and pyruvate.

**Sample**	**Concentration of inhibitor/activator**	**Predicted secondary structure elements (%)**							
		**α_1_**	**α_2_**	**α_1_ + α_2_**	**β_1_**	**β_2_**	**β_1_ + β_2_**	**Turn**	**Coil**
rAtAOX1A	Control	44	9	53	12	10	22	5	20
rAtAOX1A + SHAM	0.05 mM	36	6	42	17	12	29	7	21
	0.1 mM	36	6	42	18	12	30	7	21
	0.5 mM	37	7	44	16	11	27	7	21
rAtAOX1A + n-PG	0.05 mM	37	7	44	17	12	29	7	21
	0.1 mM	39	9	48	15	11	26	6	21
	0.5 mM	36	6	42	18	12	30	7	21
rAtAOX1A + Pyruvate	0.05 mM	37	6	43	18	12	30	7	21
	0.1 mM	36	6	42	18	12	30	7	21
	0.5 mM	36	6	42	18	12	30	7	21
	1 mM	36	6	42	18	12	30	7	21

*The data is analyzed using the CDSSTR algorithm and reference set 4*.

*Values represent the average of four to five scans*.

### SPR Kinetic Analysis of rAtAOX1A With SHAM and n-PG

The SPR technique has been extensively used to study the molecular interaction of proteins with small-molecule(s) and in drug discovery due to its improved selectivity, stability, and sensitivity (Homola, 2003; Olaru et al., 2015). The binding events of the immobilized ligand and analytes are ascertained by monitoring the changes in the SPR signal (Response unit). As the analyte is passed on an immobilized ligand through a microfluidic channel, the interaction between ligand and analyte leads to the formation of a complex structure, which causes a change in the mass on the sensor chip surface and, thereby, a change in the SPR signal of the sensorgram. This change/difference in the signal is used to derive kinetic constants for both complex formation (association) and its dissociation in a particular molecular interaction between a ligand and an analyte (Nguyen et al., 2015).

**Figure 5:**
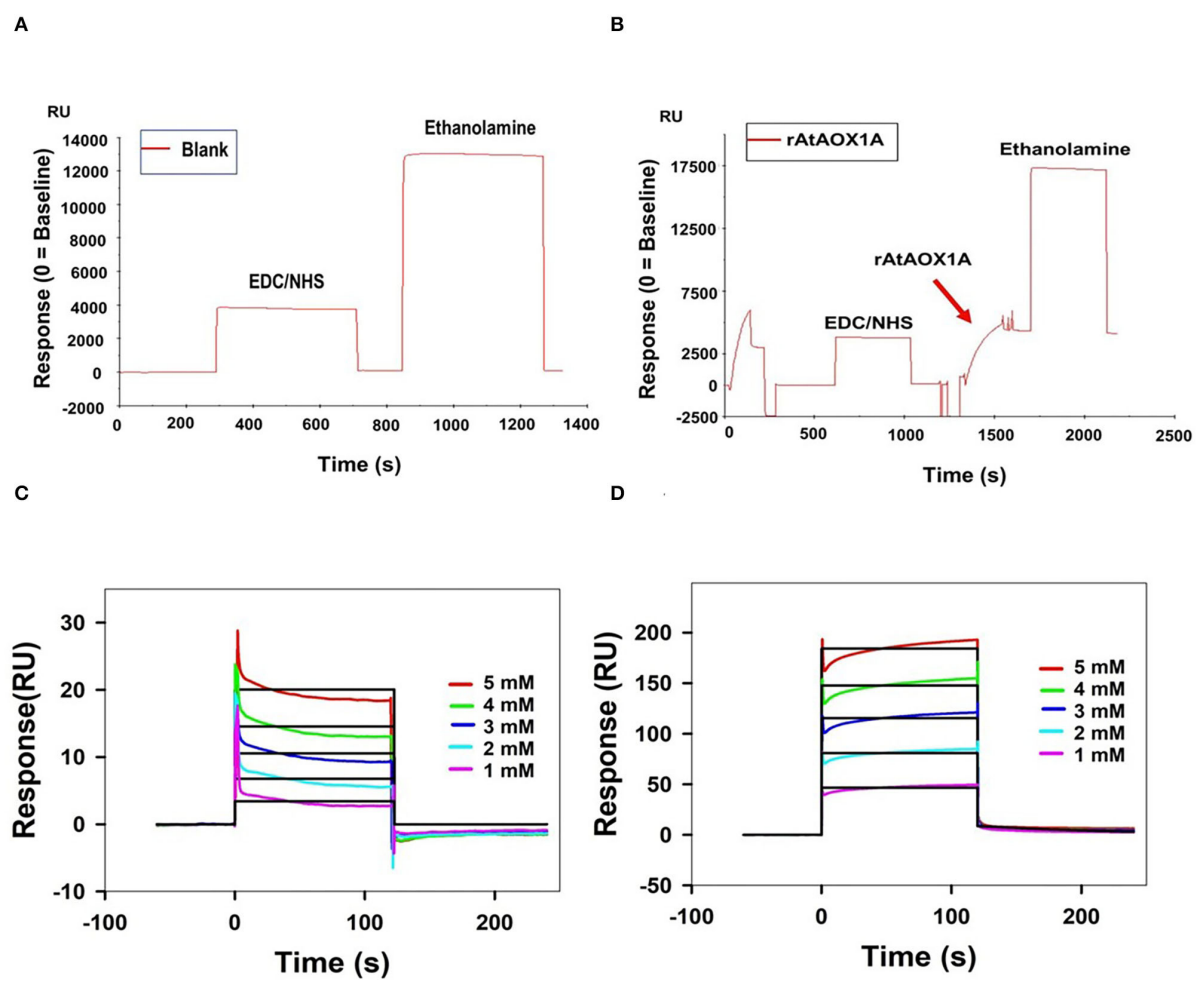
Purified rAtAOX1A is immobilized onto Series S Sensor Chip CM5. **(A)** Blank immobilization on flow cell Fc-3; **(B)** 100 μg/ml of purified rAtAOX1A (ligand) immobilization on flow cell Fc-4. Arrow indicates rAtAOX1A injection, which resulted in ~4,117 RU. SPR kinetics of SHAM and n-PG with rAtAOX1A: **(C,D)** Binding curves for SHAM and n-PG with rAtAOX1A at 25°C. The colored lines represent the concentrations of SHAM/n-PG (1, 2, 3, 4, and 5 mM), the black lines correspond to the fit lines to the data, and each fit line is a result of a global fit. The final sensorgram is representative of three cycles.

The mechanism of rAtAOX1A interaction with SHAM and n-PG is characterized by immobilization of rAtAOX1A on a CM5 sensor chip and following its binding affinities (Figure 5). Initially, the immobilization of rAtAOX1A to the Fc-4 in the sensor chip is visualized by an increase in its RU by ~4,117 when compared with its reference (blank) cell (Fc-3) RU (Figures 5A,B). Later, the analytes (SHAM and n-PG) are allowed to pass independently through both Fc-3 (reference/blank flow cell) and Fc-4 (rAtAOX1A immobilized flow cell). The binding of SHAM and n-PG with immobilized rAtAOX1A at different concentrations (1 to 5 mM) was visualized in the sensorgrams as the rate of increase in RU of Fc-4 [note: RU of blank/reference flow cell (Fc-3) corresponding analytes is subtracted automatically] (Figures 5C,D). From these sensorgrams, association, dissociation, and steady-state interaction kinetics of SHAM and n-PG with rAtAOX1A were calculated. SHAM has shown an equilibrium dissociation constant *K*_D_ = 3.08 x 10^−9^ M with the following association (*k*_a_ = 1.11 x 10^5^ M^−1^ s^−1^) and dissociation (*k*_d_ = 3.40 x 10^−4^ s^−1^) rate constants. Similarly, the kinetic parameters measured for n-PG has shown equilibrium dissociation constant *K*_D_ = 4.91 x 10^−10^ M with the following association (*k*_a_ = 1.80 x 10^7^ M^−1^ s^−1^) and dissociation (*k*_d_ = 8.85 x 10^−3^ s^−1^) rate constants. These results suggest that the n-PG (~0.49 nM) has more affinity to rAtAOX1A than SHAM (~3 nM). However, both the inhibitors bind reversibly to the rAtAOX1A, which is evident by the changes in the RU observed during their association and dissociation in corresponding sensorgrams (Figures 5C,D).

### Molecular Docking of AtAOX1A With Its Ligands

**Figure 6:**
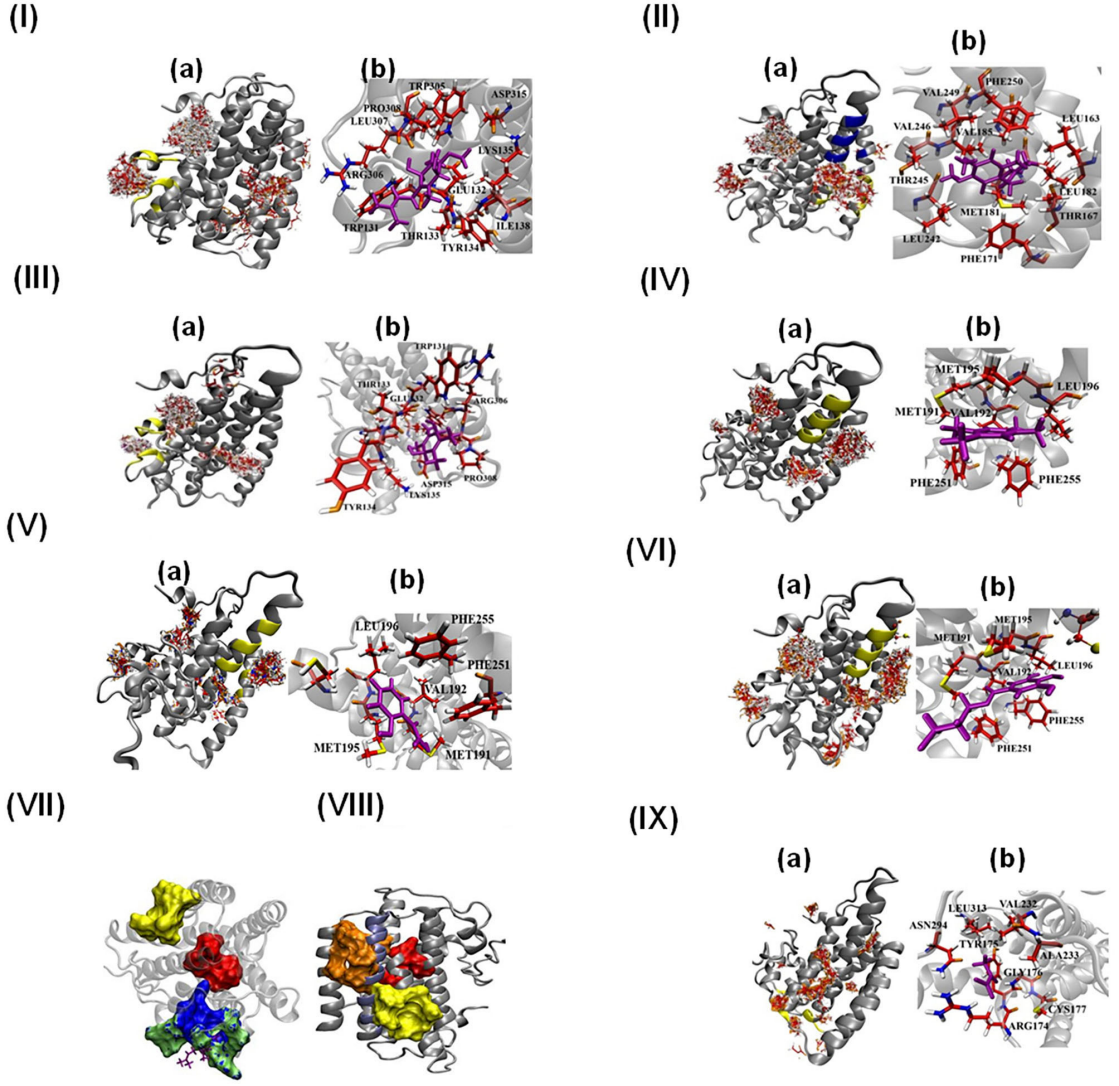
Molecular docking of AtAOX1A with different ligands **(I-VI and IX)**. (a) All possible binding pockets of a specific ligand on AtAOX1A. The yellow region on AtAOX1A secondary structure indicates the best-fit binding pocket of a particular ligand. The Blue region in panel **(IIa)** represents the inhibitor binding pocket to compare with that of UQ, and (b) residues of AtAOX1A that are involved in forming the binding pocket of the best model (Cluster 0 Element 0) according to SwissDock results. The ligands are **(I)** Q_1_H_2_, **(II)** UQ_1_, **(III)** DQH_2_, **(IV)** DQ, **(V)** SHAM, **(VI)** n-PG, **(VII)** Binding pockets of inhibitor (yellow), DQH_2_ (green surface), Q_1_H_2_ (blue surface), and diiron binding cavity (red surface). DQH_2_ is shown with purple sticks. **(VIII)** Binding pockets of inhibitor (yellow surface), ubiquinone-1 (orange surface), and diiron cavity (red surface), **(IX)** Pyruvate. Q_1_H_2_, UQ_1_, DQH_2_, DQ, SHAM, n-PG, and pyruvate are shown in purple. Color scheme: carbon (red), oxygen (orange), sulfur (yellow), nitrogen (blue), and hydrogen (white).

**Figure 7:**
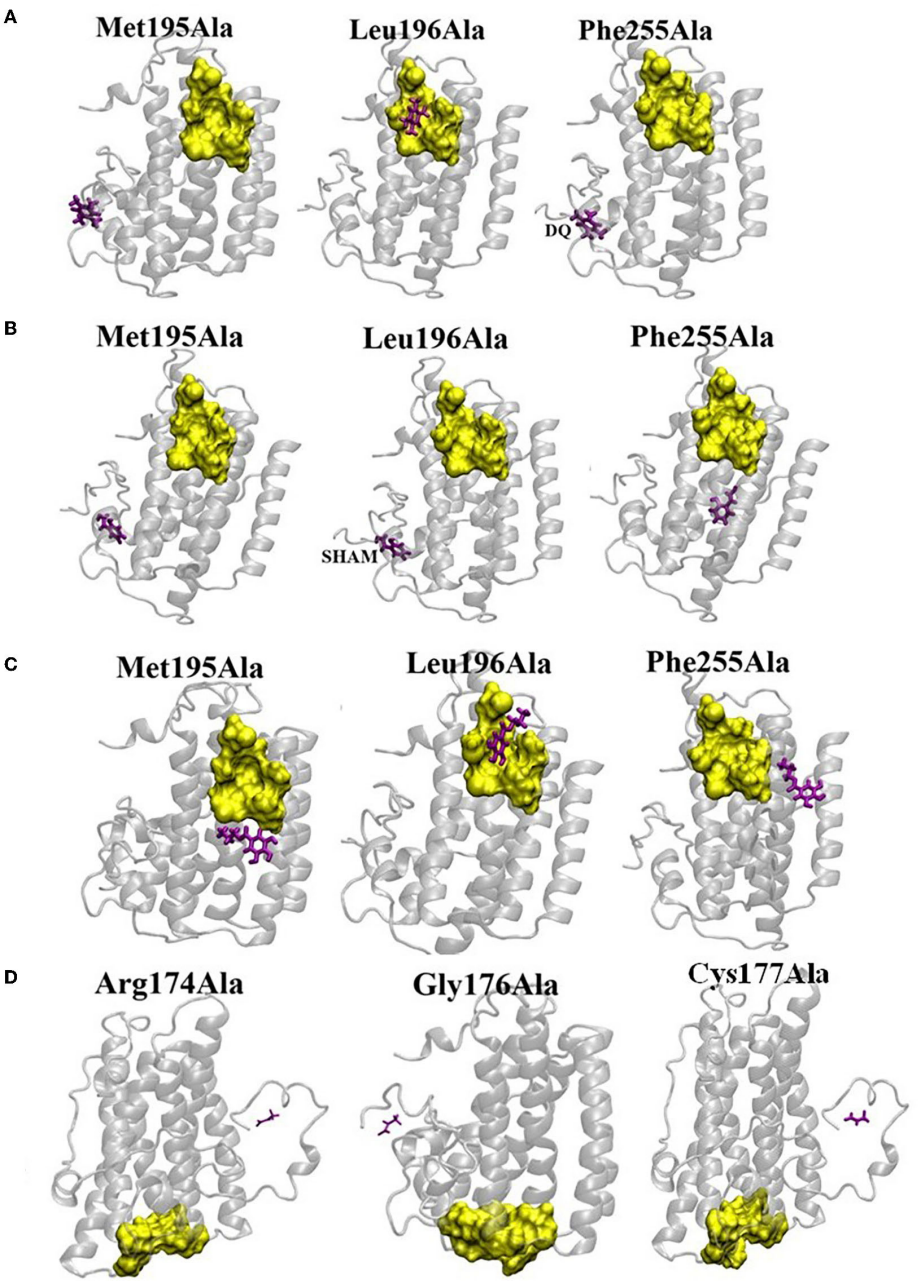
Effect of mutation on the binding affinities of DQ **(A)**, SHAM **(B)**, n-PG **(C)**, and Pyruvate **(D)**. The yellow surface indicates the location of the binding pocket in wild type (WT) AtAOX1A. The location of the ligand on the mutant AtAOX1A is shown in purple.

The interaction of the following ligands with AtAOX1A was examined by *in silico* molecular docking and mutational studies: (i) Q_1_H_2_ (reduced form of ubiquinone), which plays a significant role in the transfer of electrons from complex I and complex II of mETC to AOX and its oxidized form (UQ_1_), which is released from the AOX after the donation of electrons by Q_1_H_2_ to its catalytic center under *in vivo* conditions; (ii) DQH_2_ (reduced form of duroquinone), an analog of ubiquinol and electron donor to AOX under *in vitro* conditions, and its oxidized form (DQ) that is released from AOX after electron donation by DQH_2_ to its catalytic center; (iii) n-PG and SHAM, which inhibit the transfer of electrons through the AOX pathway, and (iv) pyruvate, which activate the AOX and, thereby, electron transfer through the AOX pathway (Figures 6, 7).

Panel (a) in Figures 6I–VI shows the most probable binding sites of the above-mentioned ligands on AtAOX1A. The Q_1_H_2_ binding site (Figure 6Ib) is composed of Trp131, Glu132, Thr133, Tyr134, Lys135, Ile138, Trp305, Arg306, Leu307, Pro308, and Asp315, while Q_1_H_2_ makes hydrogen bonds with Trp131 and Arg306, and forms a stable complex with ΔG = −7.05 kcal/mol. The ubiquinone or ubiquinone-1 (with one isoprenoid unit) binding pocket (Figure 6IIb) is made up of Leu163, Thr167, Phe171, Met181, Leu182, Val185, Leu242, Thr245, Val246, Val249, and Phe250, respectively, and the ligand is mostly hydrophobic due to the presence of five methyl groups. The binding energy of UQ_1_ in this pocket is ΔG = −6.74 kcal/mol. The binding pocket for DQH_2_ consists of Trp131, Glu132, Thr133, Tyr134, Lys135, Arg306, Pro308, and Asp315 (Figure 6IIIb). The binding energy of DQH_2_ to the AtAOX1A is ΔG = −6.63 kcal/mol. Interestingly, DQH_2_ and Q_1_H_2_ bind in the same location that is close to the diiron cavity (Figure 6VII). According to the SwissDock results, the binding pocket of DQ is made up of Met191, Val192, Met195, Leu196, Phe251, and Phe255 (Figure 6IVb). The binding energy of DQ to the AtAOX1A is ΔG = −6.11 kcal/mol. This surface exposed, hydrophobic region is located on the 2nd and 4th α-helices and is ~27Å away from the diiron center.

According to the SwissDock results, SHAM and n-PG bind to the same binding pocket (Figures 6Vb, VIb) as that of DQ. The binding energies of SHAM and n-PG are −6.01 and −6.13 kcal/mol, respectively. This observation confirms that the inhibitor binding pocket could be conserved in AtAOX1A. Figures 6VII,VIII compare the location of the diiron cavity with the inhibitor and Q_1_H_2_/DQH_2_ and UQ_1_ binding sites, respectively. It was observed that the UQ_1_ binding site bridges the diiron cavity and inhibitor binding site. Further, the pyruvate binding sites on the AtAOX1A are shown in Figure 6IXa. The most probable binding site for pyruvate (Figure 6IXb) is made of Arg174, Tyr175, Gly176, Cys177, Val232, Ala233, Asn294, and Leu313. The binding energy of pyruvate is ΔG = −6.87 kcal/mol.

### Mutational Docking Studies of AtAOX1A With Different Ligands

To identify the potent residues on AtAOX1A that may have a direct impact on the binding of DQ, SHAM, and n-PG, point mutations were performed concerning three hydrophobic residues: (i) Met195 to Ala, (ii) Leu196 to Ala, and (iii) Phe255 to Ala in the DQ/inhibitor binding pocket (Figures 7A–C). The side chains of these residues lie within 3Å from the ligand as the hydrophobic interactions between the ligand and side chains hold the ligand in its binding pocket. Interestingly, upon mutations of Met195 and Phe255 to alanine, the DQ (Figure 7A), SHAM (Figure 7B), and n-PG (Figure 7C) binding locations are drastically altered in comparison to that of the wild type AtAOX1A. However, the mutation of Leu196 did not alter the binding pocket of DQ and n-PG. Similarly, in the case of pyruvate, three mutations were performed: Arg174 to Ala, Gly176 to Ala, and Cys177 to Ala (Figure 7D). All these mutations inhibited the activator from binding to the original binding site in the wild type AtAOX1A and, thus, may play primary roles in the binding of the activator.

## Discussion

### Differential Response of *E. coli*/pET28a and *E. coli*/pAtAOX1A to Mitochondrial Inhibitors

The electron transport chain components located in the inner membrane of *E. coli* possess two terminal oxidases, *viz*., ‘cytochrome oxidase *bo'* and ‘cytochrome oxidase *bd,'* to reduce molecular oxygen to water molecules and oxidize ubiquinol (Kita et al., 1984a; Anraku and Gennis, 1987; Mogi et al., 1994; Yap et al., 2010; Borisov et al., 2011). Cytochrome oxidase *bo*, which possesses heme-copper oxidase, shares functional similarities with mitochondrial cytochrome c oxidase of higher plants and is expressed during high oxygen concentrations. In contrast, cytochrome *bd* oxidase, which lacks both copper ion and the Fe–S cluster, is known to predominate in anoxic conditions and is not homologous to any other terminal oxidases, such as heme-copper oxidoreductases or AOX (Reid and Ingledew, 1979; Kita et al., 1984b; Poole and Cook, 2000; Borisov et al., 2011). The properties of both these oxidases (*bo* and *bd*) are quite distinct from the AOX, which contains a non-heme diiron carboxylate center (Berthold et al., 2002; Berthold and Stenmark, 2003; Moore et al., 2008, 2013). Therefore, in this study, BL21(DE3) *E. coli* strain was chosen to examine *in vivo* functional expression of pAtAOX1A and robust expression of rAtAOX1A protein for purification and *in vitro* characterization. AOX is known to be resistant to cyanide and sensitive to n-PG and SHAM, while *E. coli* oxidases are found to be sensitive to cyanide but not to AOX inhibitors (Bendall and Bonner, 1971; Siedow and Girvin, 1980; Fukai et al., 1999). Thus, consistent with the properties of different oxidases, the respiratory rates of *E. coli*/pAtAOX1A are resistant to KCN and sensitive to n-PG and SHAM when compared with *E. coli*/pET28a (Figures 2A–C).

In the absence of any inhibitor, after 5 h, *E. coli*/pAtAOX1A exhibited higher growth (OD_600_ = ~1) when compared with *E. coli*/pET28a (OD_600_ = ~0.8) due to the beneficial effects rendered by AtAOX1A expression in *E. coli*/pAtAOX1A. Consequently, the growth rates of *E. coli*/pAtAOX1A are found to be resistant to KCN and sensitive to n-PG and SHAM, which is not the case with the *E. coli*/pET28a (Figures 2D–I). Thus, the differential responses observed in respiration and growth rates of *E. coli*/pET28a and *E. coli/*pAtAOX1A, upon treatment with mitochondrial inhibitors, clearly demonstrate that the rAtAOX1A expressed in *E. coli* is functionally active (Berthold, 1998). The expression of AOX from *A. thaliana* in hemA *E. coli* strain (deficient in cytochrome-mediated aerobic respiration) allowed it to grow under aerobic conditions (Kumar and Söll, 1992). Also, the expression of TAO in *E. coli* and rAtAOX1A in yeast cells has shown cyanide-insensitive and ascofuranone/SHAM-sensitive growth (Fukai et al., 1999; Nihei et al., 2003; Vishwakarma et al., 2016). Nevertheless, the results from this study suggest the concentration of n-PG to be restricted to ≤0.25 mM, as treatment with higher concentrations, leads to non-specific inhibitory effects on *E. coli* oxidase(s) and/or cellular redox enzymes, as well as hydrolases (Boyd and Beveridge, 1979; Han and Park, 2009; Figure 2F). Similarly, a high concentration of SHAM (2 mM) showed an inhibitory effect on the growth of *E. coli***/**pET28a (88%), possibly due to its non-specific effect on *E. coli* cytochrome oxidases (Figure 2H). However, the non-specific effects are exacerbated more with SHAM than n-PG. Further, the studies of Berthold (1998) reported similar results using cytochrome *bd* oxidase mutants where partial inhibition in the duroquinol oxidase activity of the *E. coli* membrane was observed with SHAM but not n-PG.

### Purification of rAtAOX1A in Its Active Form

In the present study, the rAtAOX1A is overexpressed in *E. coli* BL21(DE3) and purified from *E. coli* membranes (Figure 3A). To obtain a pure, as well as active rAtAOX1A, it is essential to maintain the following conditions during its inductions and purification procedure.

#### Supplementation of Fe^2+^ Is Essential for the Expression of Active rAtAOX1A

The studies of Minagawa et al. (1990) and Ajayi et al. (2002) have reported that iron supplementation is essential during the heterologous expression of AOX. In this study, Fe^2+^ was added to the culture medium at the time of induction along with IPTG, which resulted in an active rAtAOX1A (Table 1). However, when rAtAOX1A was purified without Fe^2+^ supplementation, its activity was not detectable, despite the presence of pyruvate in all purification steps (data not shown). This could be due to a lack of formation of the hydroxo-bridged binuclear iron center, which is involved in the reduction of molecular oxygen to water, as evident by electron paramagnetic resonance studies (Berthold et al., 2002; Moore et al., 2008). Similarly, in this study, supplementation of Fe^+3^ ion to the culture medium could not retrieve AOX in pure form (data not shown).

#### Pyruvate Supplementation Is Essential During All the Steps of Purification

Pyruvate stabilizes the active enzyme conformation, although the specific mechanism of AOX activation by pyruvate is unclear (Carré et al., 2011; Elliott et al., 2014; Xu et al., 2021). In this study, the addition of pyruvate (10 mM) in all purification steps resulted in the retrieval of active rAtAOX1A from *E. coli* membranes (Table 1). However, the AOX activity was found to be insensitive to pyruvate when examined in mitochondria isolated from thermogenic plants, such as *Arum italicum* (Hoefnagel et al., 1997), *S. guttatum* (Crichton et al., 2005), and *A. maculatum* (Ito et al., 2011).

#### Detergent Choice to Solubilize the rAtAOX1A From *E. coli* Membranes

The choice of detergent usage also plays a major role in retaining AOX activity during the process of purification. For solubilizing the recombinant trypanosomal alternative oxidase (rTAO), OG was proven to be efficient (Kido et al., 2010). However, in the case of recombinant S*auromatum guttatum* alternative oxidase (rSgAOX), OG caused a decrease in the enzyme activity, while DDM improved its activity (Elliott et al., 2014). In this study, DDM was used to solubilize rAtAOX1A from *E. coli*/pAtAOX1A membranes. The protocol used in this study resulted in a 15% recovery of rAtAOX1A. Further, the oxygen uptake activity of purified rAtAOX1A in the presence of duroquinol was found to be 3.8 μmol O_2_ min^−1^ mg^−1^ protein at pH 7.5 in Tris-HCl (Table 1). Also, the activity obtained in the present study was found to be comparable (4 μmol Q_1_${\text{H}}_{2}^{.}$ min^−1.^mg^−1^ protein) with that of rAtAOX1A, which is purified from the ΔhemA-deficient *E. coli* strain (FN102), which lacks the quinol oxidase activity for cytochrome *bo* and *bd* complexes (Xu et al., 2021). In contrast, the activity of rTAO measured as quinol oxidizing activity was found to be 207 μmol min^−1^ mg^−1^ protein (Kido et al., 2010), while rSgAOX measured as oxygen uptake activity was found to be 20 μmol min^−1^ mg^−1^ protein (Elliott et al., 2014), respectively. The lower activity levels of rAtAOX1A in the present study could be due to its origin in the non-thermogenic plant (Moore et al., 2013). The docking studies of May et al. (2017) and Xu et al. (2021) indicated that the differences in the polar residues surrounding the hydrophobic cavity of the quinol binding site might change the size of the cavity, which, in turn, may lead to the differences in the strength of attraction of quinol into the active site, and, thereby, affect the activity of AOX enzyme(s).

### Structural Characterization of Purified rAtAOX1A and Its Interaction With Inhibitors and Activator

In this study, the structure of rAtAOX1A and the changes in the corresponding structure during interaction with its inhibitory (SHAM and n-PG) and activator (pyruvate) molecules are revealed by CD spectroscopy. Besides, the docking and SPR studies identified the specific binding pockets for these molecules on AtAOX1A, and revealed their binding energies/affinities during interaction with rAtAOX1A.

Analysis of far-UV (190–260 nm) CD spectra reveals the secondary structural elements such as α-helices and β-sheets present in a protein. The CD results from the present study have revealed that the purified rAtAOX1A possessed α-helices predominantly over β-sheets in its secondary structure (Figure 4A, Table 2), and this result is consistent with the secondary structural elements of rTAO and rSgAOX (Elliott et al., 2014). Besides, the following observations in this study, such as: (i) stability in the ellipticity of rAtAOX1A to a wide range of temperatures (Figures 4C,D) and (ii) retention of its helical absorbance signal up to 67% even at temperatures as high as 90°C (Figure 4E), indicated that the thermal stability of rAOX1A isoform from a non-thermogenic plant *A. thaliana* is comparable to that of rAOX from thermogenic plant *S. guttatum* and rTAO from a parasitic protozoan (Elliott et al., 2014). Further, the retention of the helical signal even at a wide range of pH conditions indicated the stability of the rAtAOX1A to changes in pH (Figure 4B). Besides, a decrease in the α-helical content and a rise in the β-sheets in rAtAOX1A upon interaction with SHAM, n-PG, and pyruvate demonstrated the conformational changes occurring in the protein, possibly due to a rearrangement in the hydrogen bond network of the secondary structural elements (Hebia et al., 2014; Yu et al., 2021; Table 2).

Furthermore, the SPR technique, which is known to provide real-time label-free binding kinetics, is used to analyze the interaction of rAtAOX1A with its inhibitors. The kinetic data obtained for n-PG and SHAM during their interaction with rAtAOX1A fitted well with the 1:1 Langmuir model. Also, n-PG (*K*_D_ = 0.49 nM) showed higher affinity with rAtAOX1A than SHAM (*K*_D_ = 3 nM) as the *K*_D_ is inversely proportional to the binding affinity (Figures 5C,D). Also, a positive correlation was observed between the *K*_D_ values (an indicator of binding affinity) obtained in this study with the IC_50_ values (an indicator of the inhibition potency) obtained for n-PG and SHAM against rAOX from different sources (Elliott et al., 2014; May et al., 2017; Xu et al., 2021). For example, the results from the studies of Xu et al. (2021) indicated that the n-PG inhibits rAtAOX1A at a concentration of ~1.58 μM (IC_50_), while SHAM inhibits at a concentration of ~33 μM (IC_50_).

In the case of plants, the crystal structure of AOX is not yet available, so far. Therefore, in the docking studies, we used the homology model generated for AtAOX1A (PMDB Accession number: PM0080189), using the crystal structure of TAO (PDB ID: 3VV9) as a template (Pennisi et al., 2016). The studies of Pennisi et al. (2016) also predicted the protein structure for N-terminal 31 residues (residues 63–93) of AtAOX1A by *ab initio*/threading program. Thus, irrespective of the origin, AOX possessed a common structural trend of forming a four-α-helix bundle with a diiron catalytic center.

The AOX1A from *A. thaliana* contained three domains: (i) a mitochondrial recognition signal peptide domain between residues 1 and 62, which detaches from AOX after mitochondrial recognition; (ii) a predicted region in the N-terminus between residues 63 and 93; and (iii) a catalytic domain between residues 94 and 354 that is responsible for the oxidation of ubiquinol, binding of activators and inhibitors, and reduction of an oxygen molecule to water. However, the model generated by Pennisi et al. (2016) includes only the catalytic domain of AtAOX1A, and the tunnel formed by four α-helices (α2, α3, α5, and α6) in this model has two prominent hydrophobic regions: (i) a catalytic cavity consisting of conserved glutamate and histidine residues (Glu183, Glu222, Glu273, Glu324, His225, and His327) hosting the diiron center and (ii) a second region with conserved residues Arg164, Asp168, Arg178, Leu182, Ala186, Leu272, Glu275, and Ala276 connects the first hydrophobic region with the lipid bilayer facing the mitochondrial matrix. Further, the monomer-monomer binding region lies between residues 94 and 127 of each monomer. However, this choice can have the least effect on the binding affinities of SHAM and n-PG as the inhibitor binding sites are far away from the monomer-monomer interacting region. Although the Cys127 residue of each monomer is involved in disulfide bond formation during the dimeric assembly of AtAOX1A, its interaction with pyruvate is not found to be important to keeping the AOX in its activation state, which is evident by the mutational studies performed by Selinski et al. (2017).

The docking results from the present study revealed that the pyruvate binding pocket contained Cys177, along with Arg174, Tyr175, Gly176, Val232, Ala233, Asn294, and Leu313 (Figure 6IX), substantiating the involvement of second cysteine (Cys177) residue in regulating the post-translational activation of AOX during its interaction with pyruvate (Polidoros et al., 2009; Moore et al., 2013). The studies of Crichton et al. (2005) identified four potential regions in AtAOX1A that played an important role in the interaction of organic acids. The Arg174, Gly176 and Cys177 present in the pyruvate binding pocket of the present study corroborated well with the residues identified in region 2, while Asn294 matched with the “N” in the ENV motif of region 3. Further, mutational docking studies performed with Arg174Ala, Gly176Ala, and Cys177Ala in AtAOX1A identified them as potential candidates for binding to pyruvate (Figure 7D). Further, the changes observed in the secondary structural elements of AtAOX1A upon interaction with pyruvate may not allow the protein to form an inactive dimeric form and might give more access to the substrate to bind, which, in turn, could lead to the full enzyme activity (Xu et al., 2021) (Figures 4H, 6IX, 7D, Table 2).

Furthermore, the analysis of binding sites for the molecules like Q_1_H_2_, UQ_1_, DQH_2_, DQ, SHAM, and n-PG on AtAOX1A using the molecular docking method indicated that because of the structural and functional similarities between Q_1_H_2_ and DQH_2_, the binding cavities of these two ligands are found to be identical to some extent (Figures 6I,III). As observed in TAO (Shiba et al., 2013), this binding cavity is near the diiron cavity formed by four glutamate residues (Glu183, Glu222, Glu273, and Glu324), but the inhibitor binding site is far from this Q_1_H_2_ and DQH_2_ binding cavity (Figure 6VII). Further, the UQ_1_ binding site (Figure 6II) is somewhat similar to that of the inhibitor (SHAM and n-PG) binding site as both are: (i) composed of hydrophobic residues and (ii) share α-2 and α-4 helices. Our results also show that the UQ_1_ binding site is slightly away from that of the inhibitor. The inhibitor and UQ_1_ binding pockets and diiron cavity have been shown in the surface model (Figure 6VIII). Besides, DQ binding site (Figure 6IV) is similar to that of the inhibitor (SHAM and n-PG) binding site. Thus, the binding of the inhibitors to a hydrophobic groove formed by the residues Met191, Val192, Met195, Leu196, Phe251, and Phe255 in AtAOX1A might block the electron transport through the AOX pathway (Figures 6V,VI). The mutational docking studies suggest that Met195 and Phe255 of AtAOX1A are the potential candidates to bind the inhibitors (Figures 7B,C). Hence, this binding pocket could be a potential “gateway” for the oxidation-reduction process in AtAOX1A.

According to the docking studies of Shiba et al. (2013), the inhibitor AF2279OH binds to the Q_1_H_2_ binding site on TAO, while the results from the present docking studies indicated that the Q_1_H_2_/DQH_2_ binding sites are different from that of inhibitors (SHAM and n-PG) binding pocket on AtAOX1A (Figure 6VII). Nevertheless, the UQ_1_ (oxidized form of Q_1_H_2_) binding site is slightly away from that of the inhibitor (Figure 6VIII). However, the inhibitors (SHAM and n-PG) bind at the DQ (oxidized form of DQH_2_) binding site. This difference in the inhibitor binding site in TAO and AtAOX1A could be due to the difference in the amino acid sequences, where the sequence similarity between them (TAO and AtAOX1A) is only 31.04% (Xu et al., 2021).

Taken together, the results obtained from CD, SPR, and docking studies suggest that binding of SHAM or n-PG to a specific hydrophobic groove associated with α2 and α4 helices on AtAOX1A might alter its α-helical conformation, which in turn may lead to the inhibition of AOX pathway.

## Conclusion

In this study, the pAtAOX1A-transformed *E. coli* showed cyanide-resistant, as well as n-PG and SHAM-sensitive respiration, and growth characteristics, confirming the functional expression of rAtAOX1A in *E. coli* BL21(DE3) cells. CD spectroscopic analysis has revealed that the purified rAtAOX1A majorly possessed an α-helical structure that is stable against a wide range of pH (2 to 12) and temperature (4° to 90°C). These results indicate that the AOX protein remains stable in the non-thermogenic plant, even under extreme temperature(s). Further, the interaction of SHAM, n-PG, or pyruvate with the rAtAOX1A caused a significant reduction in its α-helical content while retaining its ellipticity. Besides, the *in silico* docking studies, together with mutational docking studies, revealed the following: (i) the inhibitors (SHAM and n-PG) and substrate (DQ) bind in the same hydrophobic binding site due to prominent interactions with Met195 and Phe255, (ii) Q_1_H_2_ and DQH_2_ binding sites are identical and close to the diiron center, and (iii) the activator (pyruvate) binding pocket contains Cys177, that regulates the post-translational activities in AtAOX1A. Arg174 and Gly176 also play an important role in the pyruvate interaction with AtAOX1A, (iv) UQ_1_ binding pocket connects the inhibitor binding site and diiron cavity.

## Data Availability Statement

The original contributions presented in the study are included in the article/Supplementary Material, further inquiries can be directed to the corresponding author.

## Author Contributions

KP conceived the project. TVS, DS, and AV performed the wet lab experiments and interpreted the data and wrote the manuscript. MS performed docking studies. KP and MS edited the manuscript. All authors contributed to the article and approved the submitted version.

## Funding

TVS gratefully acknowledges DBT for JRF/SRF and contingency grant [Ref. No.13/AL/241/2483 dt. 25/07/2013]. DS thanks NFST for JRF and contingency grant (202021-NFST-TEL-01185), and AV acknowledges CSIR for SRF and contingency grant.

## Conflict of Interest

The authors declare that the research was conducted in the absence of any commercial or financial relationships that could be construed as a potential conflict of interest.

## Publisher's Note

All claims expressed in this article are solely those of the authors and do not necessarily represent those of their affiliated organizations, or those of the publisher, the editors and the reviewers. Any product that may be evaluated in this article, or claim that may be made by its manufacturer, is not guaranteed or endorsed by the publisher.

## Supplementary Material

Supplementary Figure 1 Confirmation of the clone (pAtAOX1A). **(A)** colony PCR: A single colony of *E. coli* cells was used as a DNA source to amplify using *AtAOX1A* primers. Lane 1, 50-1000 bp DNA ladder; lane 2, colony 1; lane 3, colony 2; lane 4, colony 3 and lane 5, non-template control. Amplified *AtAOX1A* is indicated with an arrow. **(B)** Restriction Digestion: Plasmid from pET28a and pAtAOX1A were digested with *Eco*RI and *Xho*I restriction endonucleases. Lane 1, 0.5-10 kb DNA ladder; lane 2, pET28a vector; lane 3, pAtAOX1A vector; lane 4, restriction digested pAtAOX1A; lane 5, 50–1000 bp DNA ladder. The inserted gene (*AtAOX1A*) released is indicated with an arrow. 20 μl of the sample was loaded in each well.

Supplementary Figure 2 The Biotools display of lift spectra corresponding to the peak 1656.258 Da.

Supplementary Figure 3 Secondary structural stability analysis of rAtAOX1A. The secondary structural stability was determined between 205 to 230 nm under different conditions: **(A)** pH from 2 to 12; **(B)** Temperatures from 4 to 90°C; **(C)** Temperatures from 90 to 4°C; **(D)** CD spectra of rAtAOX1A with 0.05, 0.1, and 0.5 mM of SHAM and without inhibitor; **(E)** CD spectra of rAtAOX1A with 0.05, 0.1, and 0.5 mM of n-PG, and without inhibitor; **(F)** CD spectra of rAtAOX1A with 0.05, 0.1, 0.5, and 1 mM of pyruvate, and without activator. Concentration of purified rAtAOX1A used to obtain the CD spectrum (SHAM, n-PG, and pyruvate) was 0.4 mg/ml. The final spectrum is an average of three scans as described in the Materials and Methods section.

Supplementary Data S1 The colony PCR positive clones were sequenced (Eurofins Pvt. Ltd.) with T7 forward primer (TAATACGACTCACTATAGGG). DNA sequence obtained from clone (pAtAOX1A) was aligned with original AtAOX1A gene using ClustalW Multiple alignment programs. DNA alignment results showed 100% similarity. The highlighted region represents His-tag sequences.

Supplementary Data S2 Matrix-assisted laser desorption- time of flight (MALDI-TOF) mascot search, National Center for Biotechology Information (NCBI) database protein view, Peptide mass fingerprinting (PMF) spectra and lift spectra indicated that the recombinant protein is *Arabidopsis thaliana* AOX1A.

## Abbreviations

AOX: Alternative oxidase
CD: Circular dichroism
DDM: n-dodecyl-β-D-maltopyranoside
DQH_2_: Duroquinol or Reduced duroquinone
DQ: Duroquinone
IPTG, Isopropyl: β-D-1-thiogalactoside
*K*_D_: Equilibrium dissociation constant
MALDI-TOF: Matrix-assisted laser desorption ionization time-of-flight
mETC: Mitochondrial electron transport chain
n-PG: n-propyl gallate
OD: Optical density
OG: n-octyl-β-D-glucopyranoside
rAtAOX1A: Recombinant *Arabidopsis thaliana* alternative oxidase
rSgAOX: Recombinant S*auromatum guttatum* alternative oxidase
rTAO: Recombinant trypanosome alternative oxidase
RU: Resonance unit
SHAM: Salycylhydroxamic acid
SPR: Surface plasmon resonance
Q_1_H_2_: Ubiquinol-1 or Reduced ubiquinone
UQ_1_: Ubiquinone-1.
