# Supplementary figures and images for: Structural and Biophysical Characterization of Purified Recombinant Arabidopsis thaliana's Alternative Oxidase 1A (rAtAOX1A): Interaction With Inhibitor(s) and Activator
Source: Front Plant Sci. 2022 Jun 16;13:871208. doi: 10.3389/fpls.2022.871208 (PMC9243770; doi:10.3389/fpls.2022.871208)

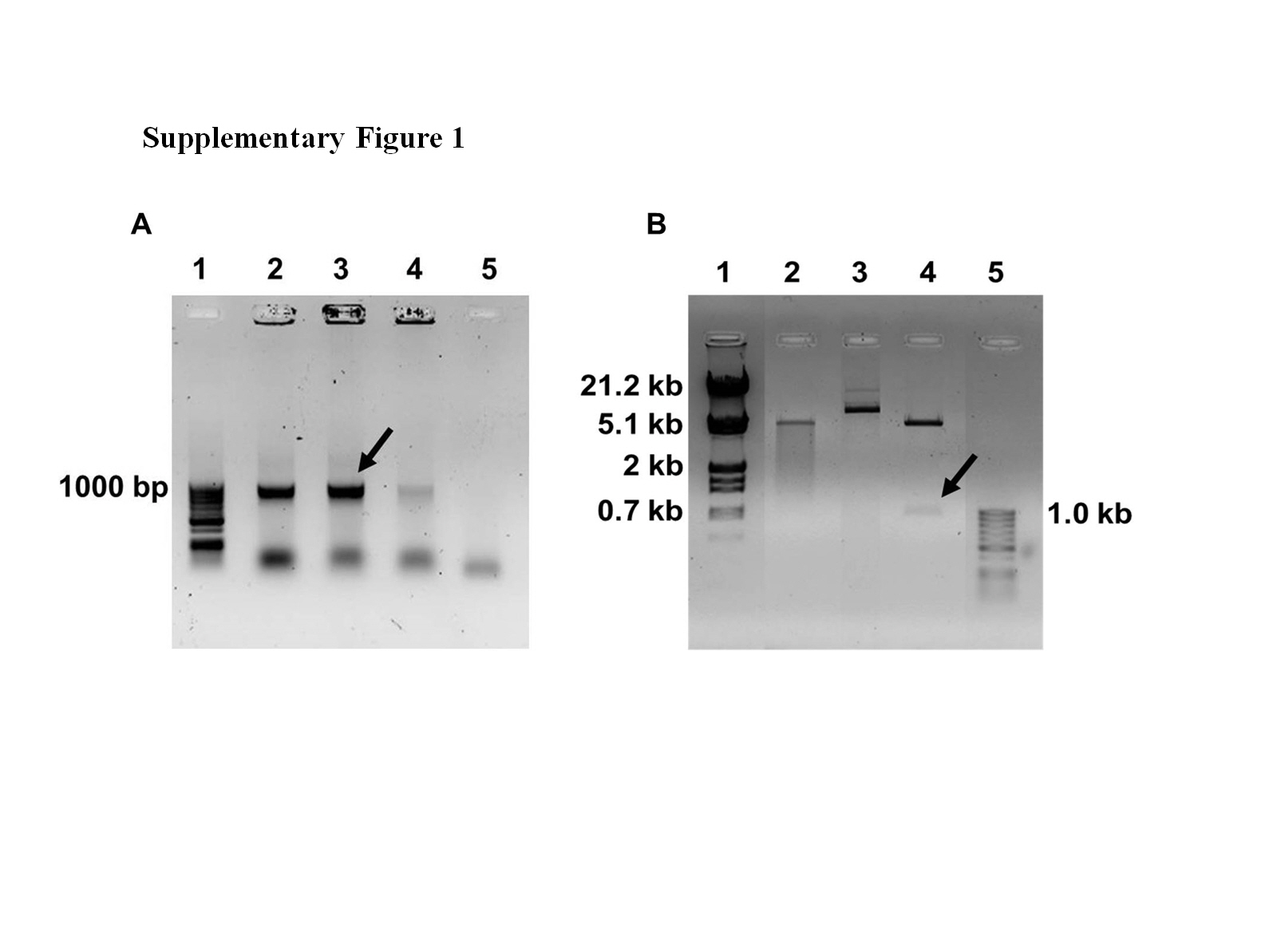

Supplement: Supplementary Figure 1 — Confirmation of the clone (pAtAOX1A). (A) colony PCR: A single colony of E. coli cells was used as a DNA source to amplify using AtAOX1A primers. Lane 1, 50-1000 bp DNA ladder; lane 2, colony 1; lane 3, colony 2; lane 4, colony 3 and lane 5, non-template control. Amplified AtAOX1A is indicated with an arrow. (B) Restriction Digestion: Plasmid from pET28a and pAtAOX1A were digested with EcoRI and XhoI restriction endonucleases. Lane 1, 0.5-10 kb DNA ladder; lane 2, pET28a vector; lane 3, pAtAOX1A vector; lane 4, restriction digested pAtAOX1A; lane 5, 50–1000 bp DNA ladder. The inserted gene (AtAOX1A) released is indicated with an arrow. 20 μl of the sample was loaded in each well. [file Image_1.jpg]

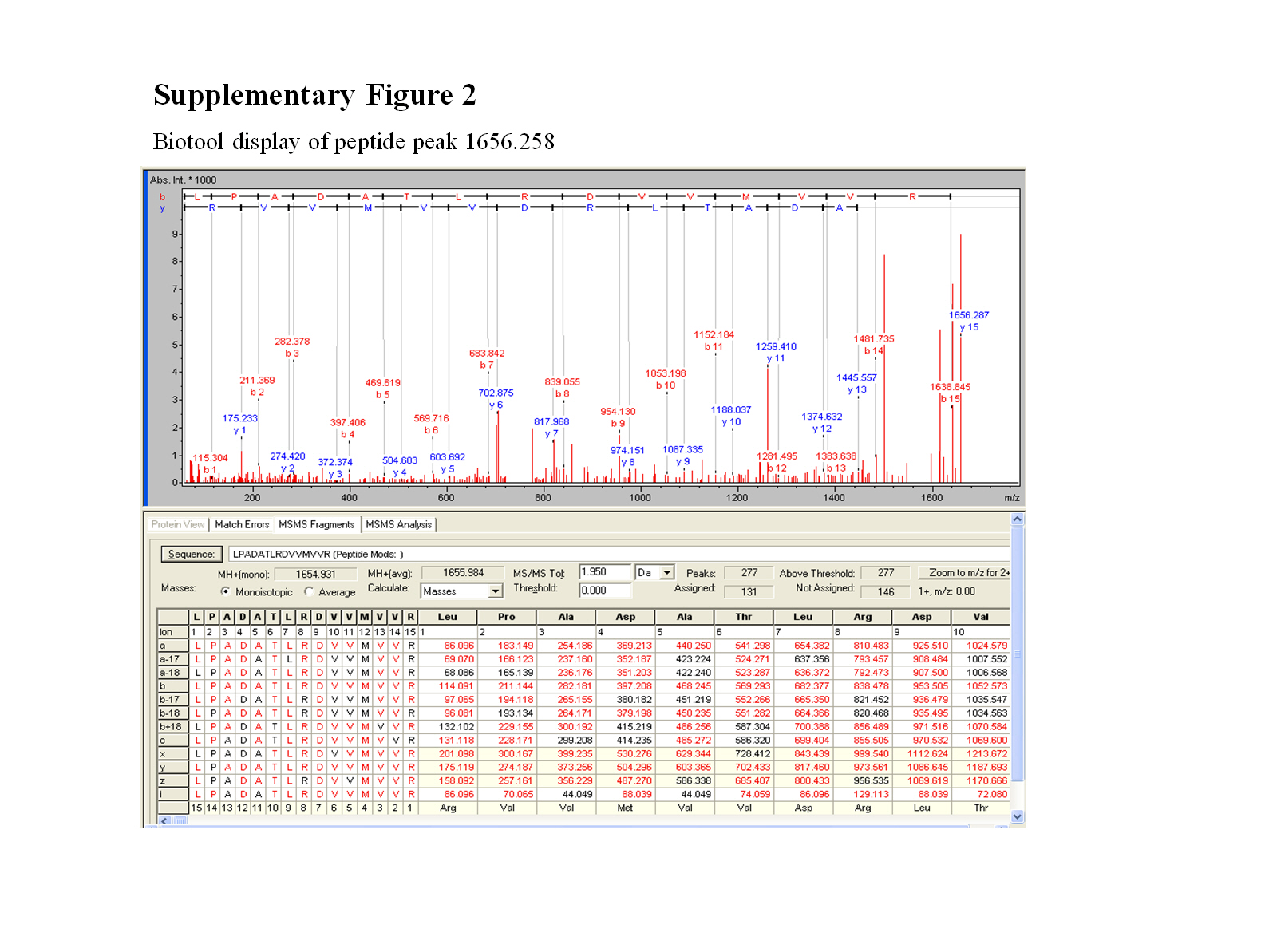

Supplement: Supplementary Figure 2 — The Biotools display of lift spectra corresponding to the peak 1656.258 Da. [file Image_2.jpg]

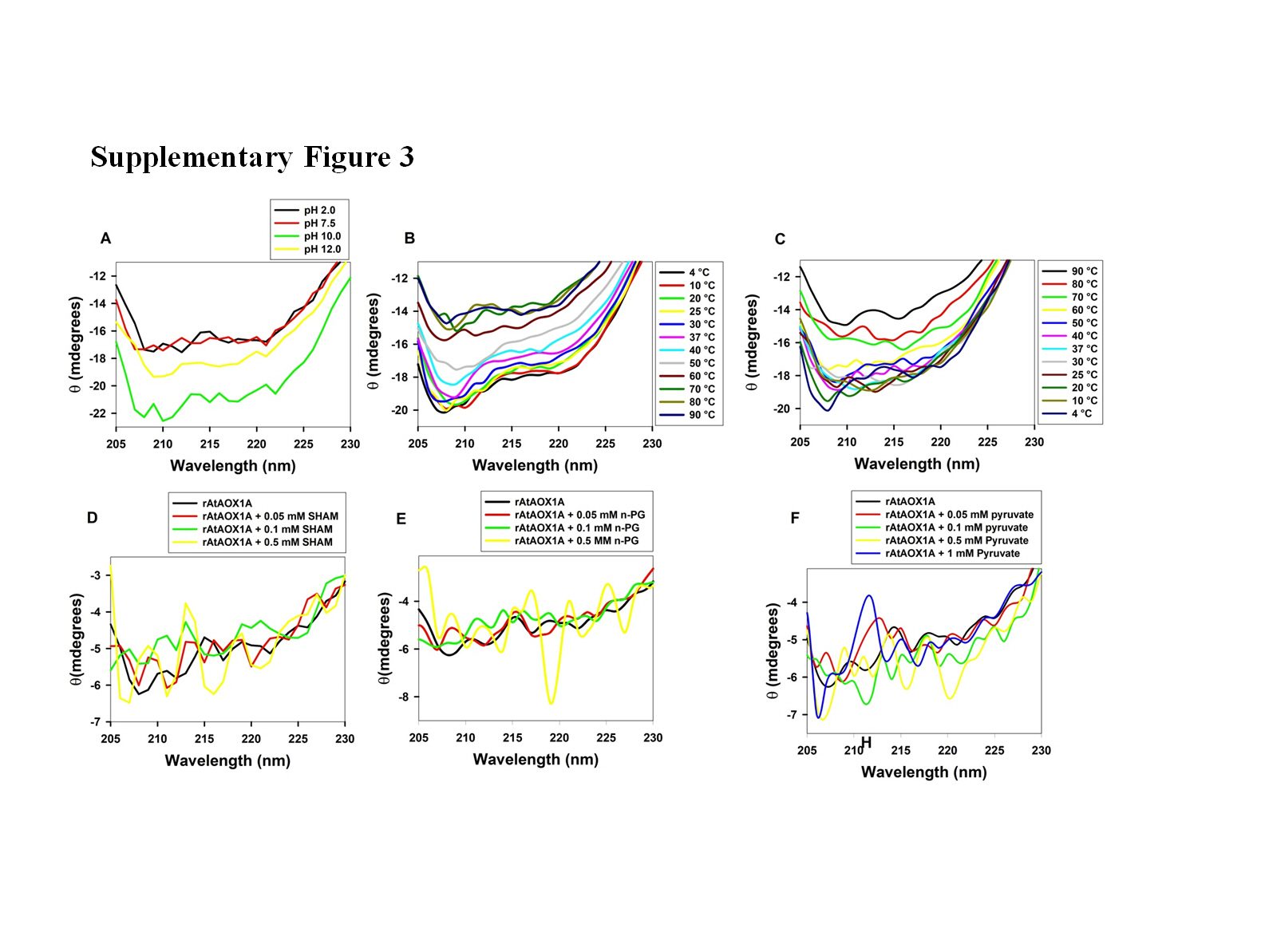

Supplement: Supplementary Figure 3 — Secondary structural stability analysis of rAtAOX1A. The secondary structural stability was determined between 205 to 230 nm under different conditions: (A) pH from 2 to 12; (B) Temperatures from 4 to 90°C; (C) Temperatures from 90 to 4°C; (D) CD spectra of rAtAOX1A with 0.05, 0.1, and 0.5 mM of SHAM and without inhibitor; (E) CD spectra of rAtAOX1A with 0.05, 0.1, and 0.5 mM of n-PG, and without inhibitor; (F) CD spectra of rAtAOX1A with 0.05, 0.1, 0.5, and 1 mM of pyruvate, and without activator. Concentration of purified rAtAOX1A used to obtain the CD spectrum (SHAM, n-PG, and pyruvate) was 0.4 mg/ml. The final spectrum is an average of three scans as described in the Materials and Methods section. [file Image_3.jpg]
