# Supplementary material for: Structural and Biophysical Characterization of Purified Recombinant Arabidopsis thaliana's Alternative Oxidase 1A (rAtAOX1A): Interaction With Inhibitor(s) and Activator
Source: Front Plant Sci. 2022 Jun 16;13:871208. doi: 10.3389/fpls.2022.871208 (PMC9243770; doi:10.3389/fpls.2022.871208)
Supplement: Supplementary Data S1 — The colony PCR positive clones were sequenced (Eurofins Pvt. Ltd.) with T7 forward primer (TAATACGACTCACTATAGGG). DNA sequence obtained from clone (pAtAOX1A) was aligned with original AtAOX1A gene using ClustalW Multiple alignment programs. DNA alignment results showed 100% similarity. The highlighted region represents His-tag sequences. [file Data_Sheet_1.pdf]

Supplementary data S1:

```
                     10        20        30        40        50        60        70        80        90       100
            ....|....|....|....|....|....|....|....|....|....|....|....|....|....|....|....|....|....|....|....|
AtAOX1A     ----------------------------------------------------------------------------------------------------
T7-Fwd seq  TTTGTGACGAACATTCCCTCTAGAAATAATTTTGTTTAACTTTAAGAAGGAGATATACCATGGGCAGCAGCCATCATCATCATCATCACAGCAGCGGCCT

                    110       120       130       140       150       160       170       180       190       200
            ....|....|....|....|....|....|....|....|....|....|....|....|....|....|....|....|....|....|....|....|
AtAOX1A     -------------------------------------------------------------------GCTAGCACGATCACTCTGGGAGAGAAAACTCCG
T7-Fwd seq  GGTGCCGCGCGGCAGCCATATGGCTAGCATGACTGGTGGACAGCAAATGGGTCGCGGATCCGAATTCGCTAGCACGATCACTCTGGGAGAGAAAACTCCG

                    210       220       230       240       250       260       270       280       290       300
            ....|....|....|....|....|....|....|....|....|....|....|....|....|....|....|....|....|....|....|....|
AtAOX1A     ATGAAGGAGGAGGACGCGAATCAGAAGAAAACAGAGAACGAATCCACCGGTGGAGACGCCGCCGGAGGTAATAACAAGGGAGATAAAGGAATCGCGAGCT
T7-Fwd seq  ATGAAGGAGGAGGACGCGAATCAGAAGAAAACAGAGAACGAATCCACCGGTGGAGACGCCGCCGGAGGTAATAACAAGGGAGATAAAGGAATCGCGAGCT

                    310       320       330       340       350       360       370       380       390       400
            ....|....|....|....|....|....|....|....|....|....|....|....|....|....|....|....|....|....|....|....|
AtAOX1A     ATTGGGGTGTTGAACCTAATAAGATTACTAAAGAAGATGGTTCTGAATGGAAGTGGAACTGTTTCAGGCCATGGGAAACGTATAAAGCTGATATAACGAT
T7-Fwd seq  ATTGGGGTGTTGAACCTAATAAGATTACTAAAGAAGATGGTTCTGAATGGAAGTGGAACTGTTTCAGGCCATGGGAAACGTATAAAGCTGATATAACGAT

                    410       420       430       440       450       460       470       480       490       500
            ....|....|....|....|....|....|....|....|....|....|....|....|....|....|....|....|....|....|....|....|
AtAOX1A     AGATCTGAAGAAGCATCATGTTCCAACGACGTTTCTTGATAGAATAGCTTATTGGACTGTTAAATCTCTTCGTTGGCCTACCGATTTGTTCTTCCAGAGG
T7-Fwd seq  AGATCTGAAGAAGCATCATGTTCCAACGACGTTTCTTGATAGAATAGCTTATTGGACTGTTAAATCTCTTCGTTGGCCTACCGATTTGTTCTTCCAGAGG

                    510       520       530       540       550       560       570       580       590       600
            ....|....|....|....|....|....|....|....|....|....|....|....|....|....|....|....|....|....|....|....|
AtAOX1A     AGATATGGATGTCGAGCTATGATGCTTGAAACTGTAGCAGCAGTACCTGGAATGGTTGGAGGAATGTTACTACACTGCAAATCGCTTCGACGTTTTGAGC
T7-Fwd seq  AGATATGGATGTCGAGCTATGATGCTTGAAACTGTAGCAGCAGTACCTGGAATGGTTGGAGGAATGTTACTACACTGCAAATCGCTTCGACGTTTTGAGC

                    610       620       630       640       650       660       670       680       690       700
            ....|....|....|....|....|....|....|....|....|....|....|....|....|....|....|....|....|....|....|....|
AtAOX1A     AAAGTGGAGGATGGATTAAGGCTCTTCTTGAGGAAGCAGAGAATGAGAGAATGCATCTTATGACATTCATGGAAGTCGCGAAACCGAAATGGTACGAGAG
T7-Fwd seq  AAAGTGGAGGATGGATTAAGGCTCTTCTTGAGGAAGCAGAGAATGAGAGAATGCATCTTATGACATTCATGGAAGTCGCGAAACCGAAATGGTACGAGAG

                    710       720       730       740       750       760       770       780       790       800
            ....|....|....|....|....|....|....|....|....|....|....|....|....|....|....|....|....|....|....|....|
AtAOX1A     AGCGCTCGTGATCACTGTGCAAGGAGTCTTCTTCAACGCTTATTTCCTTGGTTACTTAATCTCTCCCAAGTTTGCTCATCGTATGGTTGGGTACCTTGAA
T7-Fwd seq  AGCGCTCGTGATCACTGTGCAAGGAGTCTTCTTCAACGCTTATTTCCTTGGTTACTTAATCTCTCCCAAGTTTGCTCATCGTATGGTTGGGTACCTTGAA

                    810       820       830       840       850       860       870       880       890       900
            ....|....|....|....|....|....|....|....|....|....|....|....|....|....|....|....|....|....|....|....|
AtAOX1A     GAAGAAGCGATCCATTCTTATACTGAGTTTCTCAAGGAACTTGACAAAGGTAACATTGAGAATGTTCCTGCTCCGGCTATTGCTATTGATTACTGGAGGC
T7-Fwd seq  GAAGAAGCGATCCATTCTTATACTGAGTTTCTCAAGGAACTTGACAAAGGTAACATTGAGAATGTTCCTGCTCCGGCTATTGCTATTGATTACTGGAGGC

                    910       920       930       940       950       960       970       980       990      1000
            ....|....|....|....|....|....|....|....|....|....|....|....|....|....|....|....|....|....|....|....|
AtAOX1A     TTCCTGCTGATGCGACACTTCGTGATGTTGTGATGGTTGTTCGTGCTGACGAGGCTCATCACCGTGATGTAAACCATTTTGCATCTGATATTCACTACCA
T7-Fwd seq  TTCCTGCTGATGCGACACTTCGTGATGTTGTGATGGTTGTTCGTGCTGACGAGGCTCATCACCGTGATGTAAACCATTTTGCATCTGATATTCACTACCA

                   1010      1020      1030      1040      1050      1060      1070      1080      1090      1100
            ....|....|....|....|....|....|....|....|....|....|....|....|....|....|....|....|....|....|....|....|
AtAOX1A     AGGTCGTGAACTAAAGGAAGCTCCAGCTCCAATTGGGTATCATTGA------------------------------------------------------
T7-Fwd seq  AGGTCGTGAACTAAAGGAAGCTCCAGCTCCAATTGGGTATCATTGACTCGAGCACCCCACCACCACCACTGAGATCCGGCTGCTAACAAAGCCCGAAAGA

                   1110      1120      1130      1140      1150      1160      1170      1180      1190      1200
            ....|....|....|....|....|....|....|....|....|....|....|....|....|....|....|....|....|....|....|....|
AtAOX1A     ----------------------------------------------------------------------------------------------------
T7-Fwd seq  AGCTGATTTGCTGCTGCCCCGCTGAACATAACTAGCAAAACCCCTGGGGGCCCCCTAAAAGGGGCTTGAGAGGTTTTTTGGAAAGGGAAACTTACCCGTT

                   1210      1220      1230      1240      1250      1260      1270      1280      1290      1300
            ....|....|....|....|....|....|....|....|....|....|....|....|....|....|....|....|....|....|....|....|
AtAOX1A     ----------------------------------------------------------------------------------------------------
T7-Fwd seq  GGAAAGGGGACCCCCCTTTTGGGCCTTTAACCGCGGAGGGGGGGGGTTCCCCCCCCGGGCCCCCCCCTCTCCCCCCCCCCCCCCCCTCCTTTTTTTTCCC

                   1310      1320      1330      1340      1350      1360      1370      1380      1390
            ....|....|....|....|....|....|....|....|....|....|....|....|....|....|....|....|....|....|.
AtAOX1A     -------------------------------------------------------------------------------------------
T7-Fwd seq  TTTTTCACCCAAAGGGGGGGTCCCCCCAATATAAAAAGGTGGCCCTTTGTGGGGTATTTTTTTTAGAGCCCGCCACCCACAAAAAAAAAGG
```
