# Supplementary material for: Structural and Biophysical Characterization of Purified Recombinant Arabidopsis thaliana's Alternative Oxidase 1A (rAtAOX1A): Interaction With Inhibitor(s) and Activator
Source: Front Plant Sci. 2022 Jun 16;13:871208. doi: 10.3389/fpls.2022.871208 (PMC9243770; doi:10.3389/fpls.2022.871208)

Supplementary Data S2:

(i) MALDI-TOF mascot search results obtained with protein band after tryptic digestion (Figure 3A).

The pure rAtAOX1A showed a significant score of 308 and showed matching with alternative oxidase, partial [*Arabidopsis thaliana*] (gi|1872517).

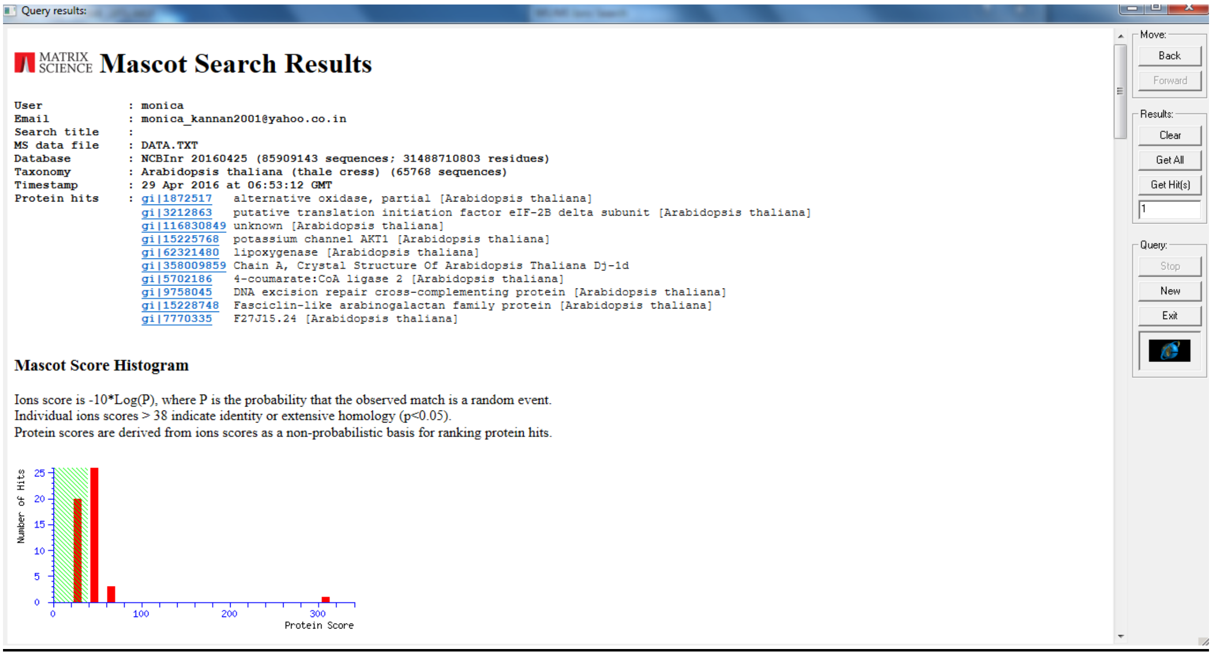

Protein identity obtained from Mascot search engine

Sl. No	Name of the protein	Acc. No	Mass	Score	Matches	Sequences
**1**	Alternative oxidase, partial [*Arabidopsis thaliana*]	gi|1872517	33123	308	6(5)	6(5)

Other possible protein matches from Mascot Search Engine

Sl. No	Name of the protein	Acc. No	Mass	Score	Matches	Sequences
2	Alternative oxidase, partial [*Arabidopsis thaliana*]	gi|4006943	40302	308	6(5)	6(5)
3	Alternative oxidase 1A [*Arabidopsis thaliana*]	gi|15228734	40125	308	6(5)	6(5)
4.	Oxidase [*Arabidopsis thaliana*]	gi|166876	35029	302	6(5)	6(5)

Protein identity obtained from Mascot search engine

Sl. No	Name of the protein	Acc. No	Mass	Score	Matches	Sequences
**1**	Alternative oxidase, partial [*Arabidopsis thaliana*]	gi|1872517	33123	308	6(5)	6(5)

MS/MS matched peptides

Query	Observed	Mr(expt)	Mr(calc)	ppm	Miss	Score	Expect	Rank	Unique	Peptide
1	1209.8541	1208.8468	1208.5979	206	0	44	0.014	1	U	R.WPTDLFFQR.R
2	1365.9727	1364.9654	1364.6990	195	1	41	0.03	4	U	R.WPTDLFFQRR.Y
3	1566.0996	1565.0923	1564.8150	177	1	23	1.7	8	U	K.SLRWPTDLFFQR.R
4	1656.2582	1655.2509	1653.9236	803	1	81	2.6e-06	1	U	R.LPADATLRDVVMVVR.A
5	2385.6287	2384.6214	2383.2172	589	1	74	2.1e-05	1	U	K.ELDKGNIENVPAPAIAIDYWR.L
6	2476.6973	2475.6900	2474.1224	634	1	45	0.015	5	U	R.ADEAHHRDVNHFASDIHYQGR.E

(ii) Over view of protein (gi|1872517) identified in Mascot search engine

Alternative oxidase, partial [*Arabidopsis thaliana*]

**Database:**	NCBInr
**Score:**	308
**Nominal mass (M_r):**	33123
**Calculated pI:**	6.29
**Taxonomy:**	*Arabidopsis thaliana*

Sequence similarity is available as an NCBI BLAST search of gi|1872517 against nr.

Search parameters

**MS data file:**	DATA.TXT
**Enzyme:**	Trypsin: cuts C-term side of KR unless next residue is P.
**Fixed modifications:**	Carbamidomethyl (C)
**Variable modifications:**	Oxidation (M)

Protein sequence coverage of six MS/MS matched peptides (bold red), of partial alternative oxidase (gi|1872517): 24%

**1**	LGEKTPMKEE	DANQKKTENE	STGGDAAGGN	NKGDKGIASY	WGVEPNKITK
**51**	EDGSEWKWNC	FRPWETYKAD	ITIDLKKHHV	PTTFLDRIAY	WTVK**SLRWPT**
**101**	**DLFFQRR**YGC	RAMMLETVAA	VPGMVGGMLL	HCKSLRRFEQ	SGGWIKALLE
**151**	EAENERMHLM	TFMEVAKPKW	YERALVITVQ	GVFFNAYFLG	YLISPKFAHR
**201**	MVGYLEEEAI	HSYTEFLK**EL**	**DKGNIENVPA**	**PAIAIDYWRL**	**PADATLRDVV**
**251**	**MVVRADEAHH**	**RDVNHFASDI**	**HYQGR**ELKEA	PAPIGYH	

Sort peptide by: Residue Number

Query	Start	End	Observed	Mr (expt)	Mr (calc)	ppm	M	Score	Expect	Rank	U	Peptide
3	95	106	1566.1	1565.092	1564.815	177	1	23	1.7	8Score > **38** indicates **identity**	U	K.SLRWPTDLFFQR.R
1	98	106	1209.854	1208.847	1208.598	206	0	44	0.014	1Score > **38** indicates **identity**	U	R.WPTDLFFQR.R
2	98	107	1365.973	1364.965	1364.699	195	1	41	0.03	4Score > **38** indicates **identity**	U	R.WPTDLFFQRR.Y
5	219	239	2385.629	2384.621	2383.217	589	1	74	2.10E-05	1Score > **40** indicates **identity**	U	K.ELDKGNIENVPAPAIAIDYWR.L
4	240	254	1656.258	1655.251	1653.924	803	1	81	2.60E-06	1Score > **38** indicates **identity**	U	R.LPADATLRDVVMVVR.A
6	255	275	2476.697	2475.69	2474.122	634	1	45	0.015	5Score > **39** indicates **identity**	U	R.ADEAHHRDVNHFASDIHYQGR.E

Details of protein accession number GI: 1872517

LOCUS AAB49302 287 aa linear
PLN 11-MAR-1997
DEFINITION Alternative oxidase, partial [*Arabidopsis thaliana*].
ACCESSION AAB49302
VERSION AAB49302.1 GI:1872517
DBSOURCE locus ATU85244 accession U85244.1
KEYWORDS
SOURCE *Arabidopsis thaliana* (thale cress)
ORGANISM *Arabidopsis thaliana*; Eukaryota; Viridiplantae; Streptophyta; Embryophyta; Tracheophyta; Spermatophyta; Magnoliophyta; eudicotyledons; Gunneridae; Pentapetalae; rosids; malvids; Brassicales; Brassicaceae; Camelineae; Arabidopsis.
REFERENCE 1(residues 1 to 287)
  AUTHORS Johnson Potter,F. and Wiskich,J.T.
  TITLE *Arabidopsis thaliana aox1* gene for alternative oxidase, partial cds
  JOURNAL Unpublished
REFERENCE 2(residues 1 to 287)
  AUTHORS Johnson Potter,F. and Wiskich,J.T.
  TITLE Direct Submission
  JOURNAL Submitted (14-JAN-1997) Botany, University of Adelaide, Adelaide, South Australia 5005, Australia
FEATURES Location/Qualifiers
source 1..287
  /organism="*Arabidopsis thaliana*"
  /db_xref="taxon:3702"
 Protein <1..287
  /product="alternative oxidase"
 Region 104..271
  /region_name="AOX"
  /note="Alternative oxidase, ferritin-like diiron-Binding domain; cd01053"
  /db_xref="CDD:153112"
 Site order(116,155,158,206,257,260)
  /site_type="other"
  /note="diiron binding motif [ion binding]"
  /db_xref="CDD:153112"
 CDS 1..287
  /gene="aox1"
  /coded_by="join(U85244.1:<1..186,U85244.1:270..398, U85244.1:698..1186,U85244.1:1268..1327)"

(iii) Search Parameters

Type of search : **MS/MS Ion Search**

Enzyme : **Trypsin**

Fixed modifications : **Carbamidomethyl (C)**

Variable modifications :**Oxidation (M)**

Mass values : **Monoisotopic**

Protein Mass : **Unrestricted**

Peptide Mass Tolerance : **± 803.5 ppm**

Fragment Mass Tolerance : **± 1.95 Da**

Max Missed Cleavages : **2**

Instrument type : **MALDI-TOF-TOF**

Number of queries : **6**

Peptide mass fingerprint (PMF) spectrum of rAtAOX1A with six major peptide peaks:

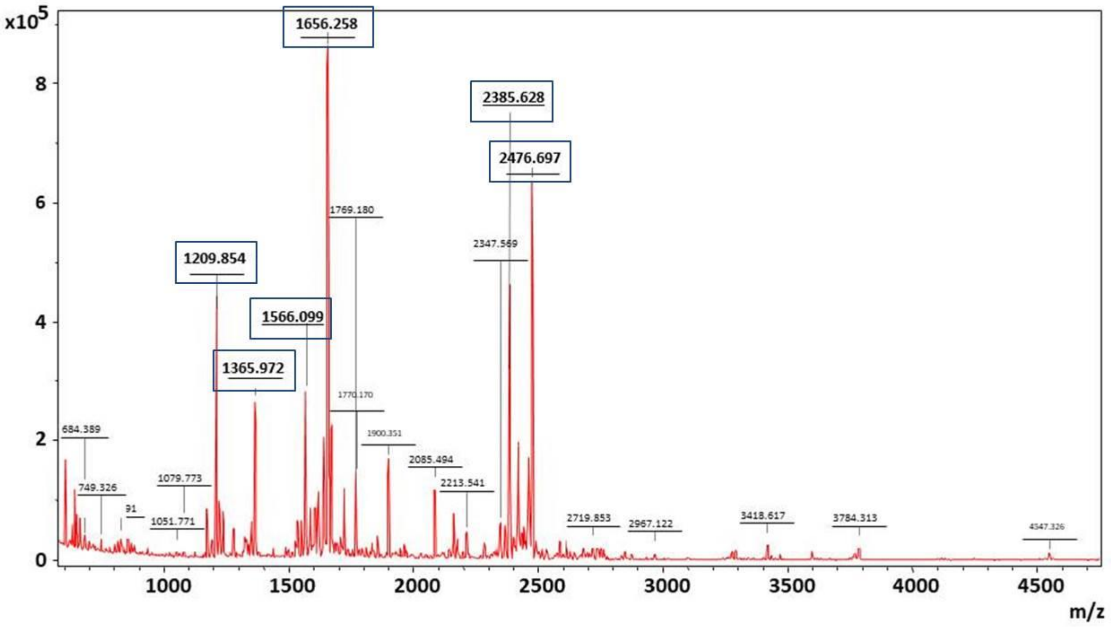

Lift spectrum for peak 1209 (m/z):

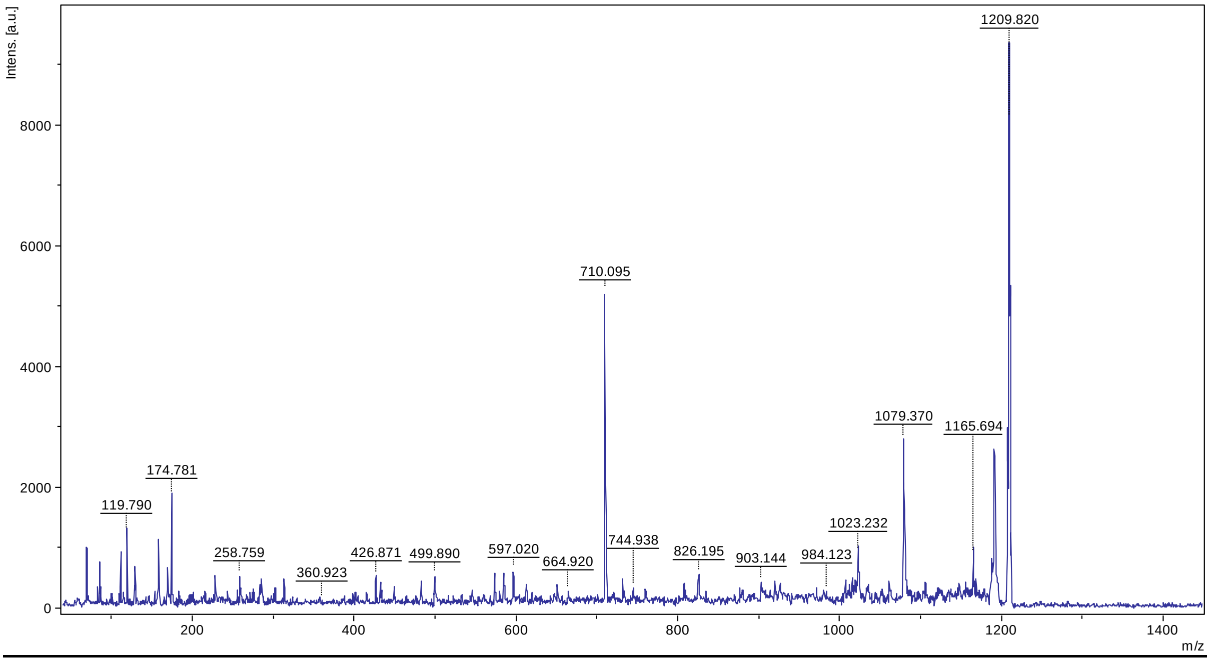

Lift spectrum for peak 1365 (m/z):

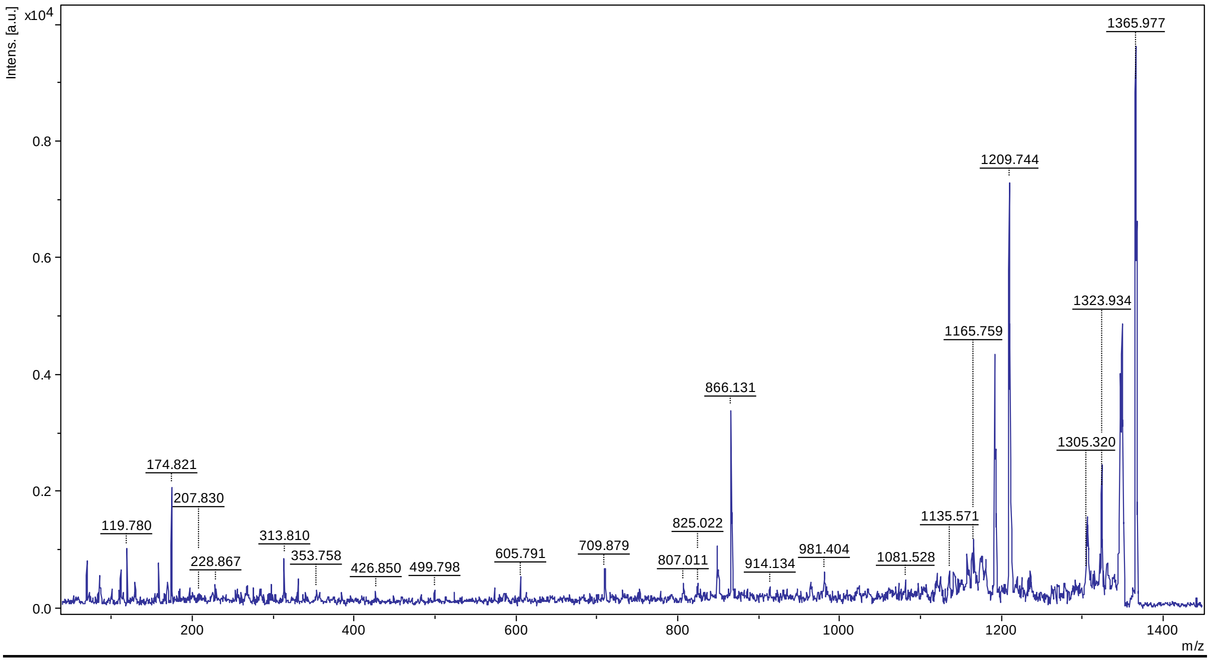

Lift spectrum for peak 1566 (m/z):

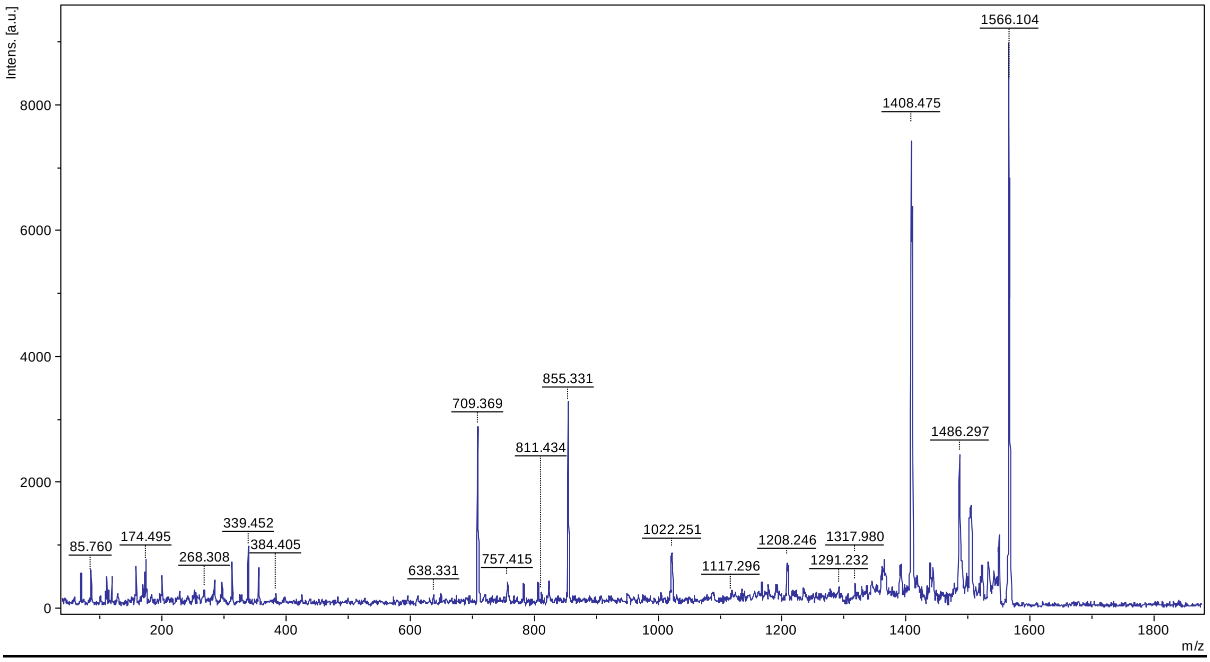

Lift spectrum for peak 1656 (m/z):

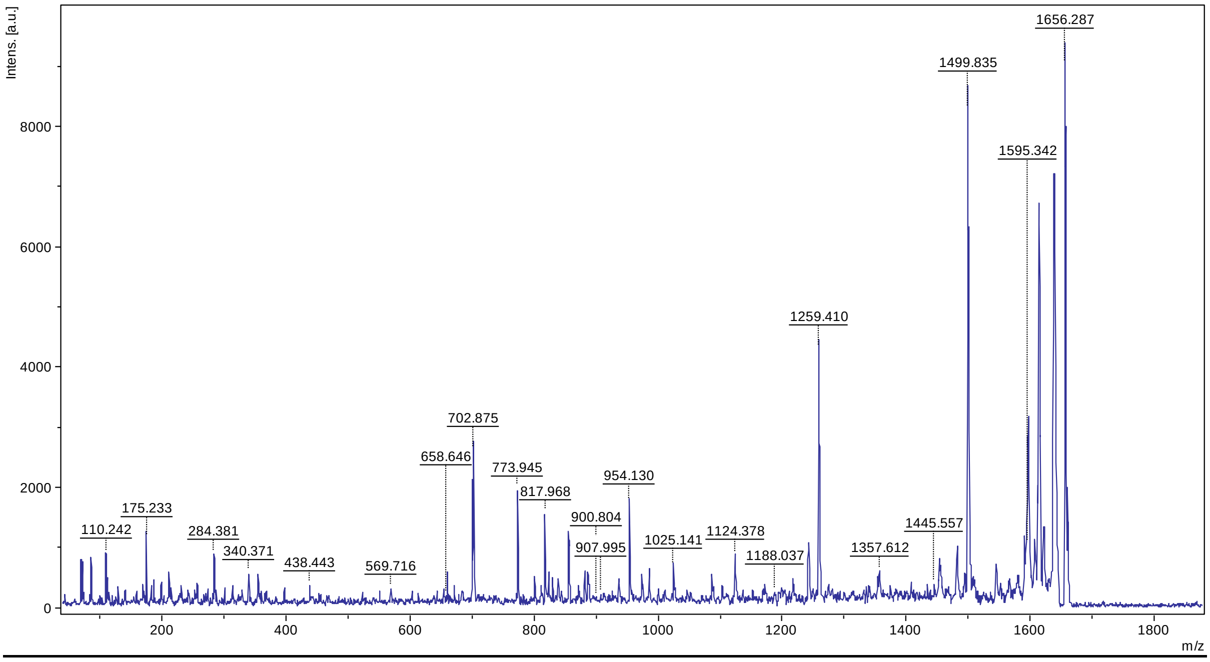

Lift spectrum for peak 2385 (m/z):

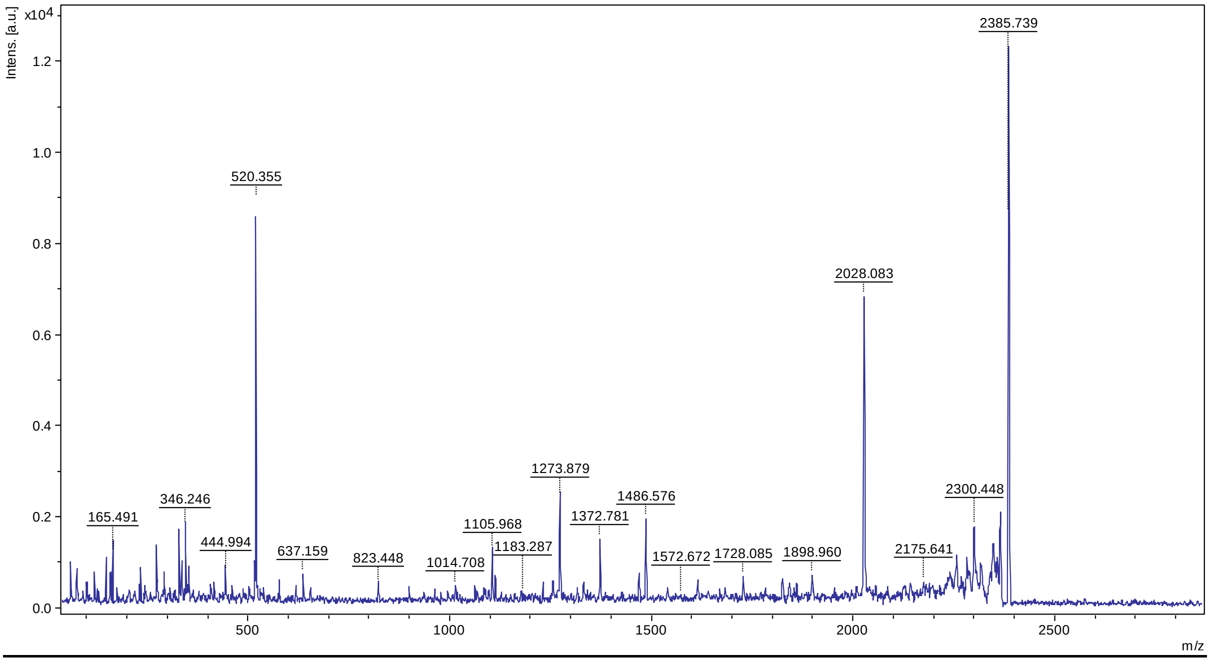

Lift spectrum for peak 2476 (m/z):

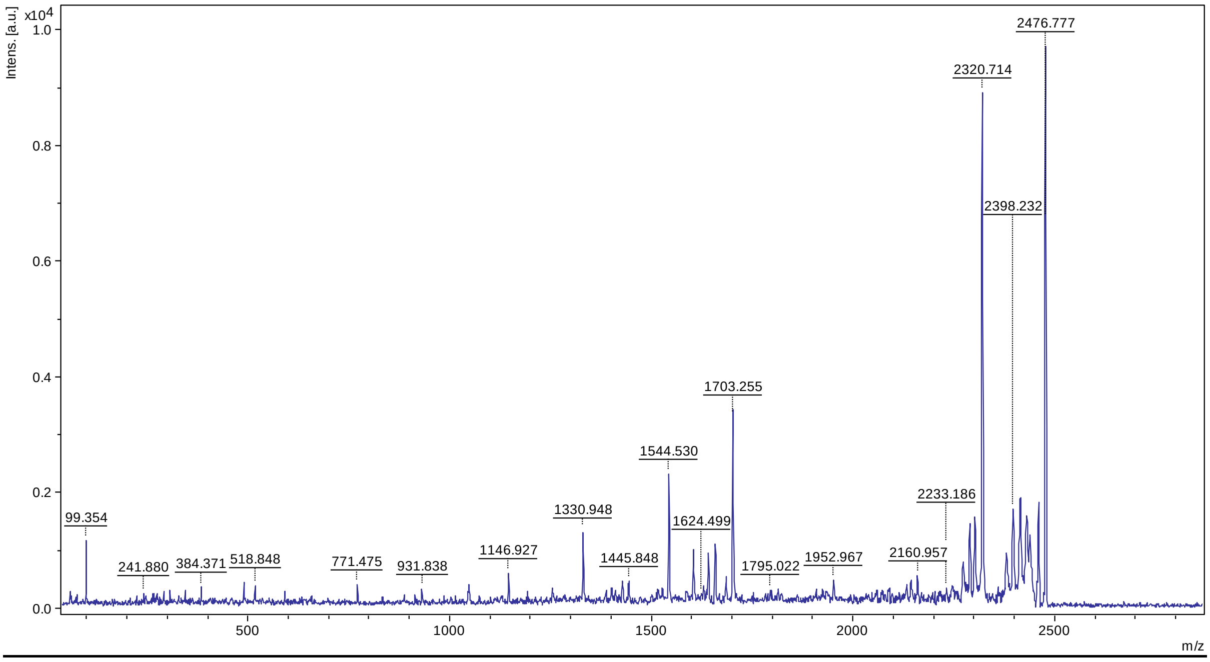

Supplement: Supplementary Data S2 — Matrix-assisted laser desorption- time of flight (MALDI-TOF) mascot search, National Center for Biotechology Information (NCBI) database protein view, Peptide mass fingerprinting (PMF) spectra and lift spectra indicated that the recombinant protein is Arabidopsis thaliana AOX1A. [file Data_Sheet_2.PDF]
